# Shift work and risk of sleep disturbances in occupational populations: a systematic review and meta-analysis

**DOI:** 10.1186/s12889-026-27636-2

**Published:** 2026-05-06

**Authors:** Ying Wang, Pei-Chao Wang, Xue-Ning Wang, Lin Yi, Dong-Liang Diao, Bing Yan

**Affiliations:** 1 School of Acupuncture-Moxibustion and Tuina, Changchun University of Chinese Medicine, Changchun, 130117 China; 2 Affiliated Hospital of Shandong University of Traditional Chinese Medicine, Jinan, 250014 China; 3 Heilongjiang University of Chinese Medicine, Harbin, 150040 China; 4 Institute of Basic Research in Clinical Medicine, China Academy of Chinese Medical Sciences, Beijing, 100700 China

**Keywords:** Shift work, Sleep disturbances, Risk factors, Occupational populations

## Abstract

**Background:**

Shift work is common worldwide and disrupts circadian rhythms, which is associated with sleep disturbances. Previous studies have often focused on single occupations or countries, limiting the generalizability of findings. A comprehensive synthesis of the association between shift work and sleep disturbances across diverse populations is lacking.

**Methods:**

We conducted a systematic search of major electronic databases up to August 15, 2025, for observational studies examining the association between shift work and sleep disturbances, using odds ratios (ORs) with 95% confidence intervals (CIs). Study quality was assessed using appropriate tools, and evidence certainty was evaluated with the GRADE framework. Heterogeneity was assessed, and pooled ORs were calculated using fixed- or random-effects models based on the level of heterogeneity. Subgroup analyses were performed by sleep disturbance type, shift type, night-shift duration, occupation, country, region, diagnostic method, and adjustment status. Publication bias was also assessed.

**Results:**

Twenty-two cross-sectional studies involving 21,677 participants from eight countries contributed 24 estimates. Shift work was associated with higher odds of sleep disturbances (OR = 1.42, 95% CI: 1.24–1.62), with comparable estimates observed after covariate adjustment (OR = 1.41, 95% CI: 1.18–1.68). Rotating shift (OR = 1.48, 95% CI: 1.23–1.77) and night shift of ≤ 8 hours (OR = 1.77, 95% CI: 1.11–2.81) were associated with higher odds of sleep disturbances. Among occupational groups, healthcare professionals showed higher odds (OR = 1.56, 95% CI: 1.21–2.01). Associations were observed across different types of sleep disturbances, with variation by geographic region and assessment method. Sensitivity analyses indicated the robustness of the estimates.

**Conclusions:**

Shift work, particularly rotating schedules and short night shifts, is robustly and consistently associated with sleep disturbances, with healthcare professionals showing comparatively higher odds. These findings underscore the need for evidence-based occupational health policies and workplace interventions designed to stabilize circadian rhythms and mitigate sleep-related risks in vulnerable populations.

**Trial registration:**

PROSPERO CRD420261285200.

**Supplementary Information:**

The online version contains supplementary material available at 10.1186/s12889-026-27636-2.

## Introduction

Shift work often involves rotating and night shifts outside typical daytime hours (7:00 a.m. to 6:00 p.m.), requiring employees to work at times misaligned with endogenous circadian rhythms [1]. The key characteristics of shift work include exposure to artificial light at night, irregular sleep-wake cycles, and misalignment between endogenous circadian rhythms and environmental cues [2, 3]. Globally, approximately 10–25% of workers engage in nonstandard schedules, particularly in the healthcare, transportation, manufacturing, and service sectors [4–6]. While such schedules are essential for maintaining 24-hour societal operations, they have been associated with a range of adverse health outcomes, including sleep disturbances, metabolic dysfunction, cardiovascular diseases, and certain cancers [1, 7–9]. Among these outcomes, sleep disturbances represent some of the most immediate and prevalent consequences, largely driven by circadian disruption [10].

Previous studies, however, report inconsistent associations between shift work and sleep disturbances. Some epidemiologic investigations have shown that rotating or night shifts increase the risk of insomnia, poor sleep quality, and excessive daytime sleepiness [11–13], whereas others observed weaker associations after adjusting for occupational factors (e.g., work schedule, occupational group, and shift characteristics) and lifestyle factors (e.g., exercise, smoking, and alcohol use) [14–16]. These discrepancies may reflect differences in study design, population characteristics, exposure definitions, and outcome measurements. In addition, prior reviews often focused on specific occupational groups, limiting generalizability.

To integrate and clarify the existing literature, we conducted a systematic review and meta-analysis to quantify the association between shift work and sleep disturbances among occupational populations and to explore potential sources of heterogeneity, thereby providing a more comprehensive synthesis of the existing evidence to inform occupational health strategies and future research.

## Materials and methods

This systematic review and meta-analysis was conducted in accordance with the Preferred Reporting Items for Systematic Reviews and Meta-Analyses (PRISMA) guidelines [17]. The protocol was prospectively registered in the International Prospective Register of Systematic Reviews (PROSPERO, CRD420261285200) and is available in the Supplementary Material 2. As this study was based on published data, ethical approval and informed consent were not required.

### Literature search strategy

We conducted a comprehensive literature search across eight electronic databases, including Web of Science, Cochrane Library, PubMed, EMBASE, China National Knowledge Infrastructure (CNKI), VIP Database for Chinese Technical Periodicals (VIP), Wanfang Data Knowledge Service Platform (Wanfang Data), and Chinese Biomedical Literature Database (CBM), from their inception to August 15, 2025. Both Medical Subject Headings (MeSH) terms and free-text keywords were used to ensure the sensitivity and specificity of the search. The main search terms included combinations of keywords related to sleep disturbances (e.g., sleep initiation dysfunction, insomnia disorder, sleeplessness, or awakening early) and shift work schedule (e.g., rotating work, night shift, shift work night, or shift work). To minimize the risk of missing eligible publications, we also manually screened the reference lists of all included studies and relevant reviews. The detailed search strategies, including the exact terms used for each database, are presented in Supplementary Tables S1-S5. No language restrictions were applied during the literature search.

### Eligibility criteria

#### Inclusion criteria

Inclusion criteria were as follows:

1. included studies in occupational populations that reported the relationship between shift work and sleep disturbances, with no restrictions on gender, age, or ethnicity;
2. adopted a cross-sectional, cohort, or case-control design;
3. defined shift work as the primary exposure, using workers who worked only daytime shifts as the reference group;
4. characterized shift work as irregular work, night shift work, rotating shift work or combinations of these patterns that disrupt circadian rhythms;
5. defined sleep disturbances broadly as nocturnal sleep problems, including self-reported poor sleep quality, insomnia disorder (e.g., difficulty initiating or maintaining sleep), short sleep duration, or clinically diagnosed sleep disorders according to international criteria (e.g., ICSD‑3-TR, DSM‑5-TR, or ICD-11) [18–20]; and.
6. reported an effect estimate for sleep disturbance outcomes, such as ORs with 95% CIs.

#### Exclusion criteria

Exclusion criteria were as follows:

1. lacked clear definitions or assessments of sleep disturbances;
2. were not peer-reviewed publications, animal experiments, case reports, personal experience articles, study protocols, reviews, data mining studies, qualitative studies, or conference abstracts;
3. did not primarily attribute sleep disturbances to shift work but rather to other factors such as social conflict or work-related stress; or.
4. had unavailable full texts or insufficient data for analysis.

### Literature selection and data extraction

Two reviewers (P-CW and X-NW) independently extracted data from all included studies, with any discrepancies resolved by adjudication from a third reviewer (LY, D-LD or YB). Data were collected using a standardized electronic extraction form, including information on author and year, country, region, research objective, occupational group, diagnostic method, study design, sample size, age, sex, OR, CI, shift type, duration of night shift, covariate adjustments, and literature quality assessment. When key data were incomplete or unclear, the corresponding authors were contacted for clarification via telephone or email.

### Assessment of literature quality

The quality of the included studies was independently assessed by two reviewers using established criteria. Cohort and Case-control studies were evaluated using the Newcastle-Ottawa Scale (NOS) [21], which examines three domains: selection, comparability, and outcome. Scores of 7–9, 4–6, and 0–3 were considered to indicate high, moderate, and low quality, respectively. Cross-sectional studies were assessed using the 11-item checklist developed by AHRQ [22], with scores of 8–11, 4–7, and 0–3 interpreted as high, moderate, and low quality, respectively. The certainty of evidence was evaluated using the GRADE approach [23].

### Statistical methods

We extracted ORs with 95% CIs from the included studies as risk estimates. For each study, ORs for sleep disturbance outcomes were obtained from logistic regression analyses, giving preference to estimates adjusted for relevant covariates to minimize potential confounding [24, 25]. An OR > 1 indicated a risk factor for sleep disturbance, whereas an OR < 1 indicated a protective factor. Heterogeneity among studies was evaluated using the I-squared (I²) statistic [26, 27]. A fixed-effects model was applied when homogeneity was observed (*P* ≥ 0.1, I² ≤ 50%), whereas a random-effects model was used in the presence of heterogeneity (*P* < 0.1, I² > 50%). To evaluate the robustness of the pooled results and identify potential sources of heterogeneity, sensitivity analyses were conducted using a leave-one-out approach [27]. Subgroup analyses were further stratified by sleep disturbance type, shift type, night-shift duration, occupation, country, region, diagnostic method and covariate adjustment. All subgroup analyses used the same modeling approach as the overall analysis to ensure consistency in addressing between-study heterogeneity. Specifically, for sleep disturbance type, outcomes were extracted from each study based on the data reported, with studies not specifying a particular type categorized as unspecified. Regions were grouped into Europe (France, Sweden, Norway, and the UK), East Asia (China, Japan, and Korea), and South America (Peru) to account for potential differences in work culture and typical shift patterns. We classified occupations into different categories based on the International Standard Classification of Occupations (ISCO-08) [28] and similarities in work characteristics related to circadian disruption. Participants were categorized as healthcare professionals (e.g., nurses, doctors, midwives, or clinical staff), general or industrial workers (e.g., manufacturing, construction, or transportation), and a mixed or others category for studies including mixed occupations or populations represented by a single study (e.g., firefighters or employees).

In this meta-analysis, shift type was defined as rotating shift work, permanent night shift, or unspecified shift work when the rotation pattern was not clearly specified. Rotating shift work referred to schedules alternating between day, evening, and/or night shifts, while permanent night shift referred to fixed work schedules during nighttime hours only. When multiple shift types were reported within one study (e.g., rotating with night shifts or permanent night shifts), data were extracted separately for each group. Furthermore, the night-shift duration was categorized as > 8 h, ≤ 8 h, or unclear if not explicitly reported. The 8-hour threshold was selected based on its alignment with standard working hours under most national and international labor regulations [29, 30]. For subgroups with at least two studies, pooled effect sizes and I² statistics were calculated, whereas subgroups with only a single study were summarized narratively.

Publication bias was assessed using funnel plots and Egger’s test [31]. A *P*-value < 0.05 was considered statistically significant. All statistical analyses were conducted using Stata version 18.0 (StataCorp, College Station, TX, USA). Data and code are available in Tables 1 and 2 and Supplementary File 1.

**Table 1:** Characteristics of cross-sectional studies (Part 1)

Study	OR	LCI	UCI	Sample size	Male /Female	Age (yrs)^a^	Adjustments^b^	Literature quality assessment	
								AHRQ score	Quality level
Niedhammer et al. 1994 [32]	1.98	1.12	3.51	220	0/220	≤ 24, 25–34 or ≥ 35	NA	7	Moderate quality
	1.01	0.43	2.33	49	0/49				
Åkerstedt et al. 2002 [33]	1.56	1.36	1.79	2289	NA	16–84	NA	8	High quality
Ursin et al. 2009 [14]	1.3	1.0	1.6	2044	842/1202	40–45	work situation, education, family income, marriage status, urban/rural living, subjective health, exercise, smoking, and alcohol use	8	High quality
Lin et al. 2012 [34]	2.26	1.57	3.28	769	0/769	20–45	age, duration of employment, marital status, number of children, and medical center	8	High quality
Øyane et al. 2013 [35]	1.48	1.1	1.99	1315	121/1194	32.4 (32.0-32.8)	age, gender, and years of work experience, marital status and children living at home	8	High quality
Ma et al. 2016 [36]	1.283	0.842	1.956	1160	NA	≥ 18	NA	10	High quality
Voinescu et al. 2018 [37]	1.4	0.6	3.2	40	27/13	18–65	gender, age, marital status, level of education, body mass index (kg/m^2^ ), length in service, number of hours worked per day, and number of days worked per week	9	High quality
Ma et al. 2018 [38]	0.74	0.42	1.31	67	49/18	19–65	sex, age, ethnicity, education status, married statu, smoking status, environmental smoking status, physical exercise, nap times, mental health	9	High quality
	1.37	1.07	1.74	381	324/57				
Uekata et al. 2019 [39]	1.54	0.98	2.41	223	0/223	30.0 (25.0–41.0)	age, body mass index, smoking, habitual drinking, menstruation, premenstrual syndrome, and the presence of a spouse	9	High quality
Li et al. 2019 [40]	1.984	1.175	3.351	635	NA	18–40	NA	10	High quality
Zheng et al. 2019 [41]	2.15	1.66	2.8	2175	NA	NA	NA	9	High quality
Caballero-Alvarado et al. 2020 [42]	3.13	1.01	9.73	33	NA	NA	NA	9	High quality
Dong et al. 2020 [43]	2.018	0.918	4.437	1179	NA	NA	NA	11	High quality
Jang et al. 2020 [44]	0.858	0.536	1.374	1188	NA	< 40, 40–49 or ≥ 50	age, sex, BMI, education, income, marital status, smoking, alcohol consumption, caffeine intake, exercise, fatigue, depression, anxiety, PTSD, type of job, employment period, work schedule, frequency of emergency events and off-duty work	7	Moderate quality
Li et al. 2020 [45]	1.21	1.08	1.37	3428	NA	< 30, 30–49 or ≥ 50	NA	9	High quality
Zhang et al. 2020 [46]	3.11	1.57	6.19	87	NA	< 30, 30–59 or ≥ 60	gender, age, ethnicity, education, marital status, monthly income, type of work, length of service, smoking status, alcohol consumption and physical exercise	8	High quality
Liu et al. 2021 [47]	1.958	1.12	3.488	589	0/589	≤ 40	NA	8	High quality
Zhao et al. 2022 [48]	1.37	1.11	1.69	309	NA	18–60	demographic characteristics (such as gender, age, and monthly income), occupation, and lifestyles	9	High quality
Jiang et al. 2022 [49]	0.997	0.835	1.19	1883	1129/754	33.46 ± 7.40	NA	8	High quality
Pan et al. 2024 [50]	1.073	0.408	2.823	106	NA	≤ 40 or > 40	NA	9	High quality
Liu et al. 2024 [51]	2.05	1.173	3.617	159	NA	≤ 60	NA	8	High quality
He et al. 2025 [52]	0.691	0.513	0.932	1,349	NA	< 25, 25–44 or ≥ 45	NA	8	High quality

*OR* Odds ratio, *LCI* Lower 95% confidence interval, *UCI* Upper 95% confidence interval, *NA* Not available

^a^Average age at baseline, or the highest mean when the study presented the population in stratified groups, with SD or range presented in parentheses; age categories are reported as available

^b^Adjustments refers to covariates included in multivariable regression models as reported in the original studies to control for potential confounding factors

**Table 2:** Characteristics of cross-sectional studies (Part 2)

Study	Sleep disturbance type	Country	Region^a^	Research objective	Occupation^b^	Diagnostic method	Study design	Shift type^c^	Shift characteristics	Night-shift duration^d^
Niedhammer et al. 1994 [32]	Unspecified	France	Europe	Nurses	Healthcare professional	Clinical diagnosis	Cross-sectional study	Rotating shift	Alternating with nights: 06: 00–14: 00 / 13: 00–21: 00 / 20: 00–06: 00	> 8 h
								Permanent night shift	Permanent night shift: 20: 00–06: 00	> 8 h
Åkerstedt et al. 2002 [33]	Unspecified	Sweden	Europe	Socioeconomic groups	Mixed/Others	Clinical diagnosis	Cross-sectional study	Rotating shift	NA	Unclear
Ursin et al. 2009 [14]	Insomnia disorder	Norway	Europe	Workers	General/industrial workers	Karolinska Sleep Questionnaire	Cross-sectional study	Unspecified shift work	NA	Unclear
Lin et al. 2012 [34]	Poor sleep quality	China	East Asia	Nurses	Healthcare professional	PSQI (> 5)	Cross-sectional study	Rotating shift	Rotation shift: day shift (08: 00–16: 00), evening shift (16: 00–24: 00 or 14: 00–22: 00), and night shift (00: 00–08: 00)	≤ 8 h
Øyane et al. 2013 [35]	Insomnia disorder	Sweden	Europe	Nurses	Healthcare professional	BIS	Cross-sectional study	Permanent night shift	NA	Unclear
Ma et al. 2016 [36]	Poor sleep quality	China	East Asia	Doctors and nurses	Healthcare professional	PSQI (≥ 8)	Cross-sectional study	Permanent night shift	NA	Unclear
Voinescu et al. 2018 [37]	Insomnia disorder	the UK	Europe	Workers	General/industrial workers	Basic Nordic Sleep Questionnaire	Cross-sectional study	Rotating shift	Rotating shifts: rotating work- working day and night shifts (after 19: 00) in rotation	Unclear
Ma et al. 2018 [38]	Poor sleep quality	China	East Asia	Workers	General/industrial workers	PSQI (> 5)	Cross-sectional study	Permanent night shift	Fixed night shift: 18:00–08:00	> 8 h
								Rotating shift	Two-shift: 08: 00–20: 00 and 20: 00–08: 00	> 8 h
Uekata et al. 2019 [39]	Poor sleep quality	Japan	East Asia	Nurses and midwives	Healthcare professional	PSQI (≥ 6)	Cross-sectional study	Rotating shift	Rotating 12.5 h night shifts	> 8 h
Li et al. 2019 [40]	Poor sleep quality	China	East Asia	Clinical staff	Healthcare professional	PSQI (> 7)	Cross-sectional study	Rotating shift	NA	Unclear
Zheng et al. 2019 [41]	Poor sleep quality	China	East Asia	Workers	General/industrial workers	PSQI (≥ 6)	Cross-sectional study	Rotating shift	Shift work: 16: 00–24: 00 / 24: 00–08: 00	≤ 8 h
Caballero-Alvarado et al. 2020 [42]	Insomnia disorder	Peru	South America	Doctors and nurses	Healthcare professional	AIS (≥ 6)	Cross-sectional study	Permanent night shift	NA	Unclear
Dong et al. 2020 [43]	Poor sleep quality	China	East Asia	Nurses	Healthcare professional	PSQI (> 5)	Cross-sectional study	Rotating shift	Night shift frequency per month: 1–3	Unclear
Jang et al. 2020 [44]	Insomnia disorder	Korea	East Asia	Firefighters	Mixed/Others	ISI (> 7)	Cross-sectional study	Rotating shift	9-day cycle: three daytime shifts followed by three 12 h night shifts (18: 00–09: 00) and each night shift is followed by 1 rest day	> 8 h
Li et al. 2020 [45]	Insomnia disorder	China	East Asia	Workers	General/industrial workers	AIS (≥ 6)	Cross-sectional study	Rotating shift	the day-shift worker is defined as a person working day shift at 8: 00–16: 00, evening shift at 16: 00–24: 00, or night shift at 24: 00–8: 00 of the following day	≤ 8 h
Zhang et al. 2020 [46]	Unspecified	China	East Asia	Workers	General/industrial workers	PSQI (> 7)	Cross-sectional study	Rotating shift	Two shifts: a day shift and a night shift, 12 h per shift	> 8 h
Liu et al. 2021 [47]	Unspecified	China	East Asia	Clinical staff	Healthcare professional	PSQI (> 7)	Cross-sectional study	Rotating shift	NA	Unclear
Zhao et al. 2022 [48]	Insomnia disorder	China	East Asia	employees	Mixed/Others	Nakata’s Self-Management Sleep Questionnaire	Cross-sectional study	Unspecified shift work	NA	Unclear
Jiang et al. 2022 [49]	Insomnia disorder	China	East Asia	Workers	General/industrial workers	PSQI	Cross-sectional study	Rotating shift	the daytime shift is from 8: 00–18: 00, and the night shift is from 18: 00– 8: 00, with 24 h of rest between each shift	> 8 h
Pan et al. 2024 [50]	Poor sleep quality	China	East Asia	Nurses	Healthcare professional	PSQI (> 5)	Cross-sectional study	Permanent night shift	NA	Unclear
Liu et al. 2024 [51]	Poor sleep quality	China	East Asia	Medical staff	Healthcare professional	PSQI (≥ 7)	Cross-sectional study	Permanent night shift	NA	Unclear
He et al. 2025 [52]	Poor sleep quality	China	East Asia	Midwives	Healthcare professional	PSQI (> 7)	Cross-sectional study	Rotating shift	Day shift with regular night shift: fixed rotation cycle of 1–2 days of day shifts followed by one night shift, and then 1–3 days off	Unclear

*PSQI* Pittsburgh Sleep Quality Index, *BIS* Bergen Insomnia Scale, *AIS* Athens Insomnia Scale, *ISI* Insomnia Severity Index

^a^Region refers to the geographic grouping of studies, used to account for potential differences in work culture and typical shift patterns

^b^Research objective was classified into different categories according to the International Standard Classification of Occupations (ISCO-08)

^c^Shift type was defined as rotating shift work, permanent night shift, or unspecified shift work when the rotation pattern was not clearly specified

^d^Night-shift duration was categorized as > 8 h, ≤ 8 h, or unclear if not explicitly reported

## Results

**Fig. 1:**
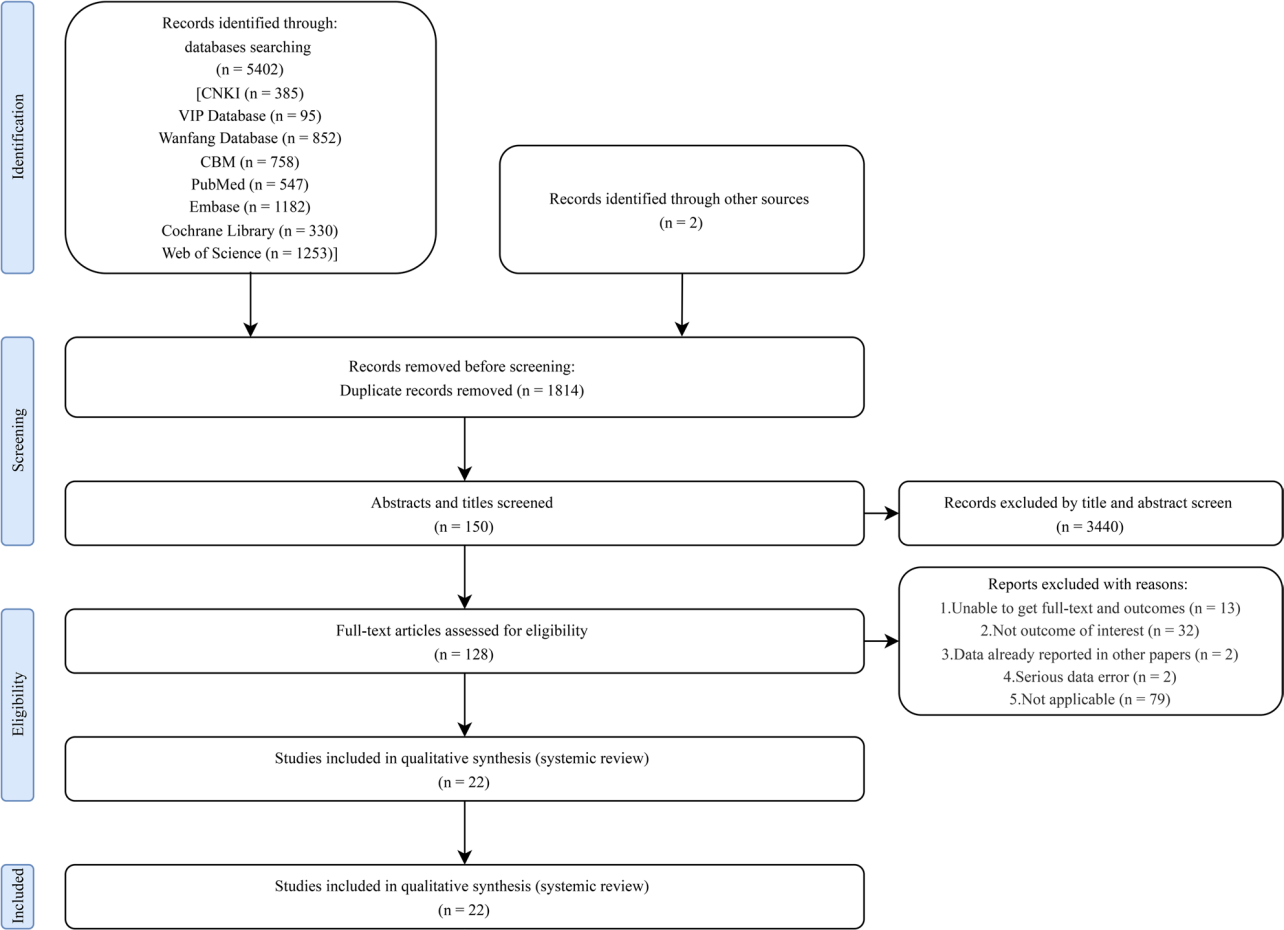
PRISMA flow diagram of study selection for the systematic review and network meta-analysis

The results of the systematic review are presented in Fig. 1. We identified 5,402 records through database searches and 2 additional records from reference lists of included studies and relevant reviews. After removing 1,814 duplicates, titles and abstracts were screened, and 128 full-text articles were assessed for eligibility. Ultimately, 22 studies met the inclusion criteria and were included in the qualitative synthesis, contributing a total of 24 distinct estimates of the association between shift work and sleep disturbance outcomes.

### Study characteristics and results of the methodological quality assessment

This meta-analysis included 22 cross-sectional studies comprising 21,677 participants from eight countries, contributing 24 data groups for analysis [14, 32–52]. Sleep disturbances included insomnia disorder (*n* = 8) and poor sleep quality (*n* = 10). The majority of studies (*n* = 16) were conducted in East Asia, primarily in China (*n* = 14), with one study each from Japan and Korea. Five studies were conducted in Europe (Sweden = 2, France = 1, Norway = 1 and the UK = 1) and one in South America (Peru = 1).

The majority of the studies (*n* = 11) involved healthcare professionals, while the remainder included general or industrial workers (*n* = 8) and mixed or other occupations (*n* = 3). Sleep disturbances were primarily assessed using the Pittsburgh Sleep Quality Index (PSQI), with the Athens Insomnia Scale (AIS), the Bergen Insomnia Scale (BIS), and other validated tools used less frequently. Among shift types, rotating shift work was most commonly reported (*n* = 13), permanent night shift was investigated in seven studies, and two studies did not specify the rotation pattern. The night-shift duration was reported in nine studies, most exceeding 8 h (*n* = 6). Nine studies reported adjustments for common covariates, such as demographic factors, lifestyle behaviors, and mental health status. The sample size of individual studies ranged from 33 to 3,428, with most participants being working-age adults. Twelve studies provided only broad age categories or did not report age, four enrolled exclusively female participants, and nine reported sex distribution.

All included studies were cross-sectional and assessed for methodological quality using the 11-item AHRQ checklist. The AHRQ scores ranged from 7 to 11, indicating moderate to high overall quality. Specifically, two studies were rated as moderate quality (score 7), while twenty studies were rated as high quality (scores 8–11). No study was rated low. Details of study characteristics and individual quality assessment are presented in Tables 1 and 2.

### Risk of bias assessment

As more than 10 studies were included in the meta-analysis, publication bias was assessed using Egger’s test and funnel plots. Egger’s test indicated no significant small-study effects (*P* = 0.275), and visual inspection of the funnel plot revealed no notable asymmetry, suggesting a low likelihood of publication bias (Supplementary Figures S1–S2 and Table S6).

### Results of the meta-analysis

#### Relationship between shift work and sleep disturbances

**Fig. 2:**
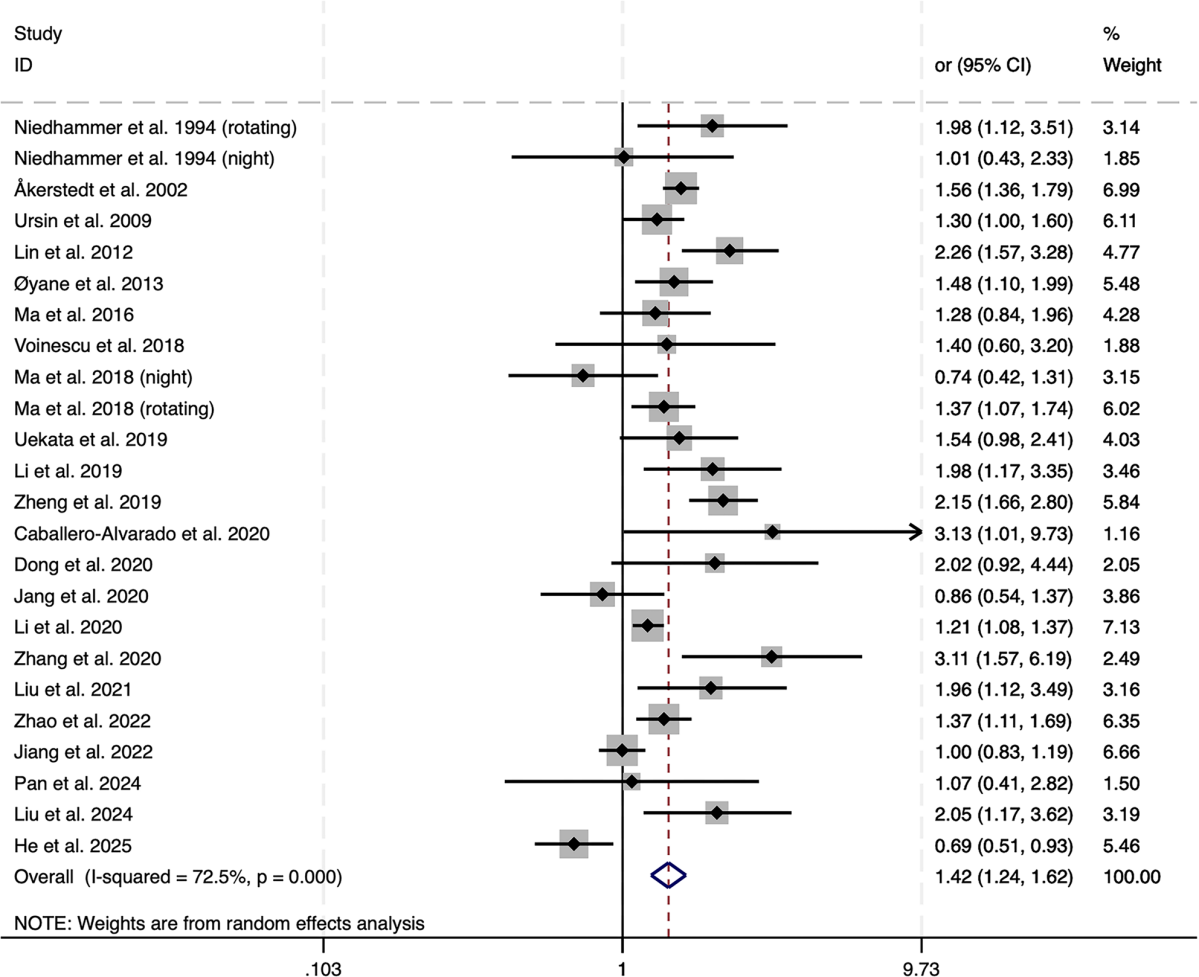
Meta-analysis of association of shift work and sleep disturbances. *Note.* OR = odds ratio; CI = confidence interval

A total of 22 studies involving occupational populations were included to assess the association between shift work and sleep disturbances. Substantial heterogeneity was observed across studies (I² = 72.5%), and thus a random-effects model was applied. As shown in Fig. 2, the pooled OR was 1.42 (95% CI: 1.24–1.62). Leave-one-out sensitivity analysis confirmed the robustness of the findings, with no single study materially affecting the pooled estimate.

#### Subgroup analyses of shift work by sleep disturbance type

**Fig. 3:**
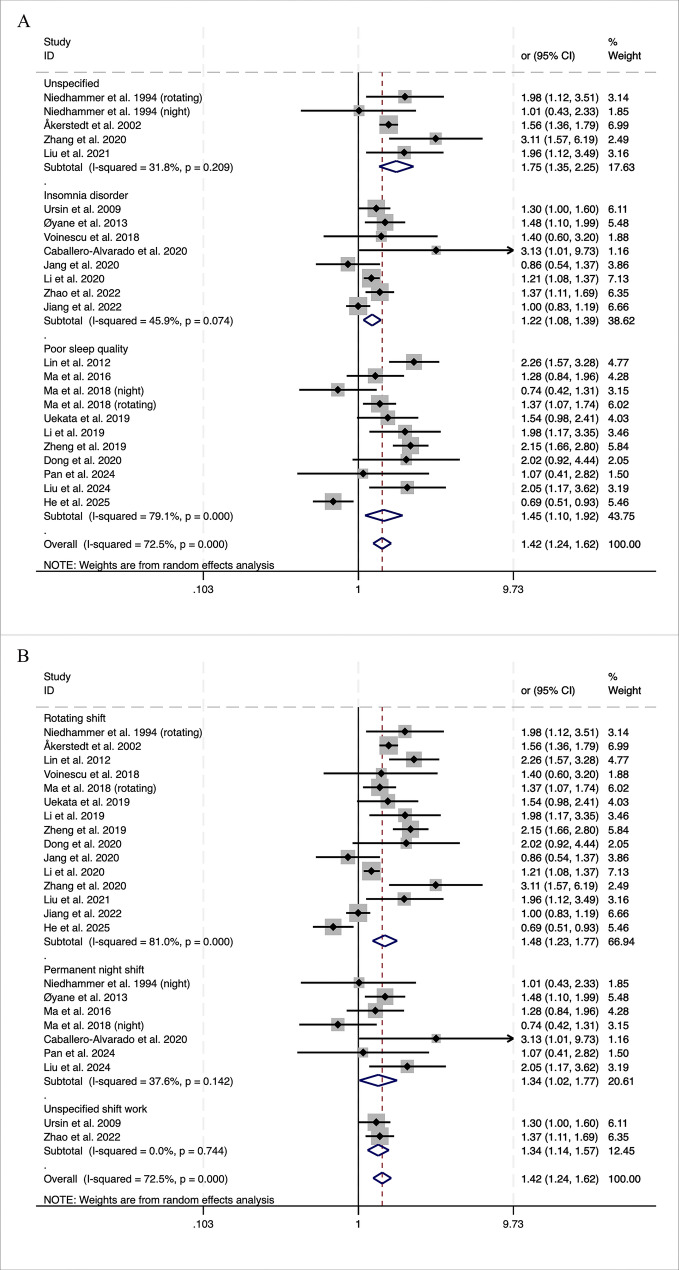
Subgroup analyses of the association between shift work and sleep disturbances by sleep disturbance type (**a**) and shift type (**b**). Note. OR = odds ratio; CI = confidence interval

When stratified by sleep disturbance type, increased odds were observed across all categories (Fig. 3a). Studies reporting unspecified sleep disturbances showed the highest OR (1.75, 95% CI: 1.35–2.25) with moderate heterogeneity (I² = 31.8%). For insomnia disorder, the pooled OR was 1.22 (95% CI: 1.08–1.39; I² = 45.9%), and for poor sleep quality, the pooled OR was 1.45 (95% CI: 1.10–1.92), with substantial heterogeneity (I² = 79.1%).

#### Subgroup analyses of shift work and sleep disturbances by shift type

Analyses stratified by shift type revealed notable differences in the association between shift work and sleep disturbances (Fig. 3b). Rotating shift work was associated with the highest odds of sleep disturbances (OR = 1.48, 95% CI: 1.23–1.77), followed by permanent night shift work (OR = 1.34, 95% CI: 1.02–1.77). Studies that did not specify shift type showed a smaller OR (1.34, 95% CI: 1.14–1.57). Heterogeneity was high in the rotating shift work subgroup (I² = 81.0%), moderate in the permanent night work subgroup (I² = 37.6%), and negligible in the unspecified subgroup (I² = 0.0%).

#### Subgroup analyses of shift work and sleep disturbances by night-shift duration

**Fig. 4:**
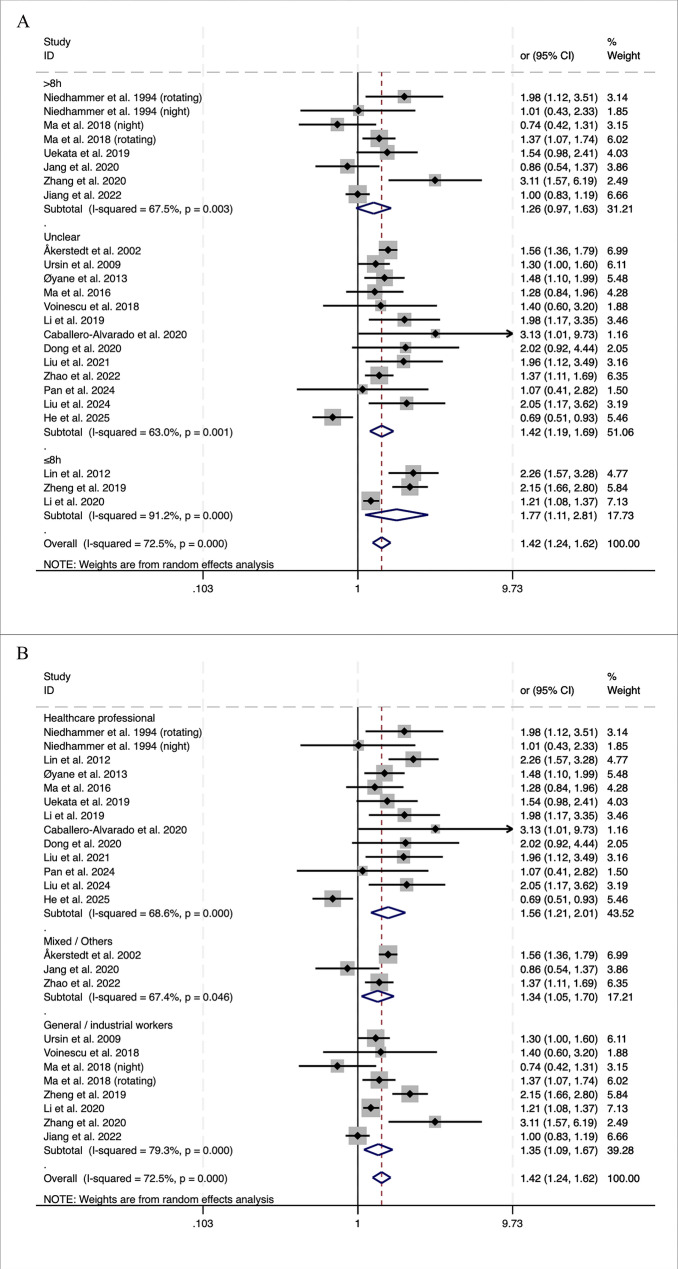
Subgroup analyses of the association between shift work and sleep disturbances by night-shift duration (**a**) and occupation (**b**). *Note.* OR = odds ratio; CI = confidence interval

Subgroup analysis by night-shift duration showed variability in effect estimates (Fig. 4a). Shorter night shifts (≤ 8 h) were associated with higher odds of sleep disturbances (OR = 1.77, 95% CI: 1.11–2.81), whereas longer shifts (> 8 h) showed a lower estimate (OR = 1.26, 95% CI: 0.97–1.63). Studies with unclear night-shift duration reported an OR of 1.42 (95% CI: 1.19–1.69). Substantial heterogeneity was observed across the ≤ 8 h, > 8 h, and unclear-duration subgroups (I² = 91.2%, 67.5%, and 63.0%, respectively).

#### Subgroup analyses of shift work and sleep disturbances by occupation

Across occupational subgroups (Fig. 4b), shift work was associated with increased odds of sleep disturbances. Healthcare professionals exhibited the strongest association (OR = 1.56, 95% CI: 1.21–2.01), followed by general or industrial workers (OR = 1.35, 95% CI: 1.09–1.67) and mixed or other occupations (OR = 1.34, 95% CI: 1.05–1.70). Heterogeneity varied among groups (I² = 68.6%, 79.3%, 67.4%).

#### Subgroup analyses of shift work and sleep disturbances by country

**Fig. 5:**
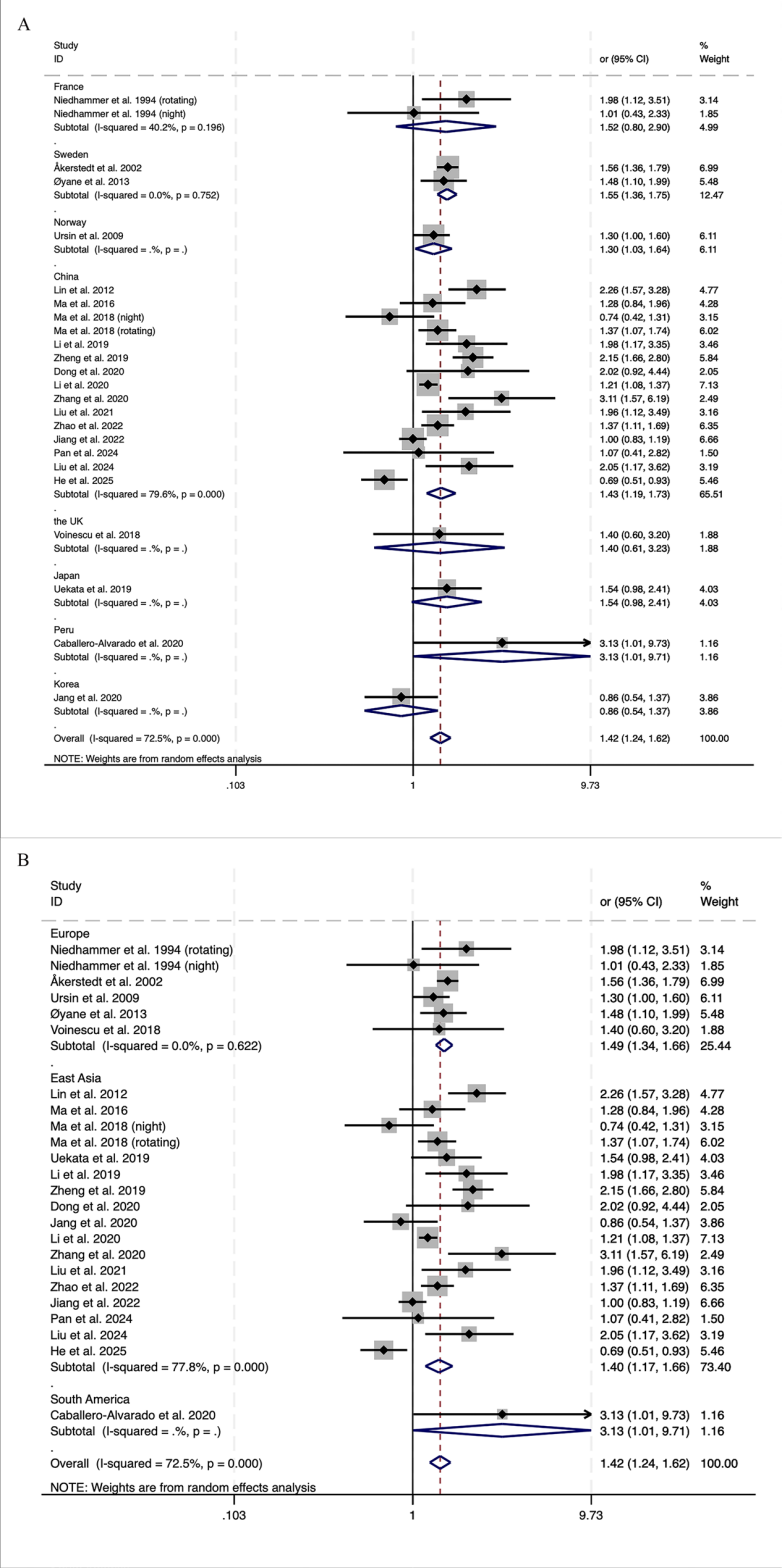
Subgroup analyses of the association between shift work and sleep disturbances by country (**a**) and region (**b**). *Note.* OR = odds ratio; CI = confidence interval

Country-specific analyses demonstrated variability in effect estimates (Fig. 5a). The pooled OR was 1.43 (95% CI: 1.19–1.73) for studies conducted in China, 1.55 (95% CI: 1.36–1.75) for Sweden, and 1.52 (95% CI: 0.80–2.90) for France. Heterogeneity was high in China (I² = 79.6%), but low in Sweden (I² = 0.0%) and France (I² = 40.2%). For countries represented by a single study (Peru, Norway, the UK, Japan, and Korea), effect estimates should be interpreted cautiously.

#### Subgroup analyses of shift work and sleep disturbances by region

Regional subgroup analyses showed differences in pooled estimates (Fig. 5b). In Europe, the pooled OR was 1.49 (95% CI: 1.34–1.66) with no observed heterogeneity (I² = 0.0%). In East Asia, the pooled OR was 1.40 (95% CI: 1.17–1.66), accompanied by substantial heterogeneity (I² = 77.8%). The single South American study from Peru reported an OR of 3.13 (95% CI: 1.01–9.73).

#### Subgroup analyses of shift work and sleep disturbances by diagnostic method

**Fig. 6:**
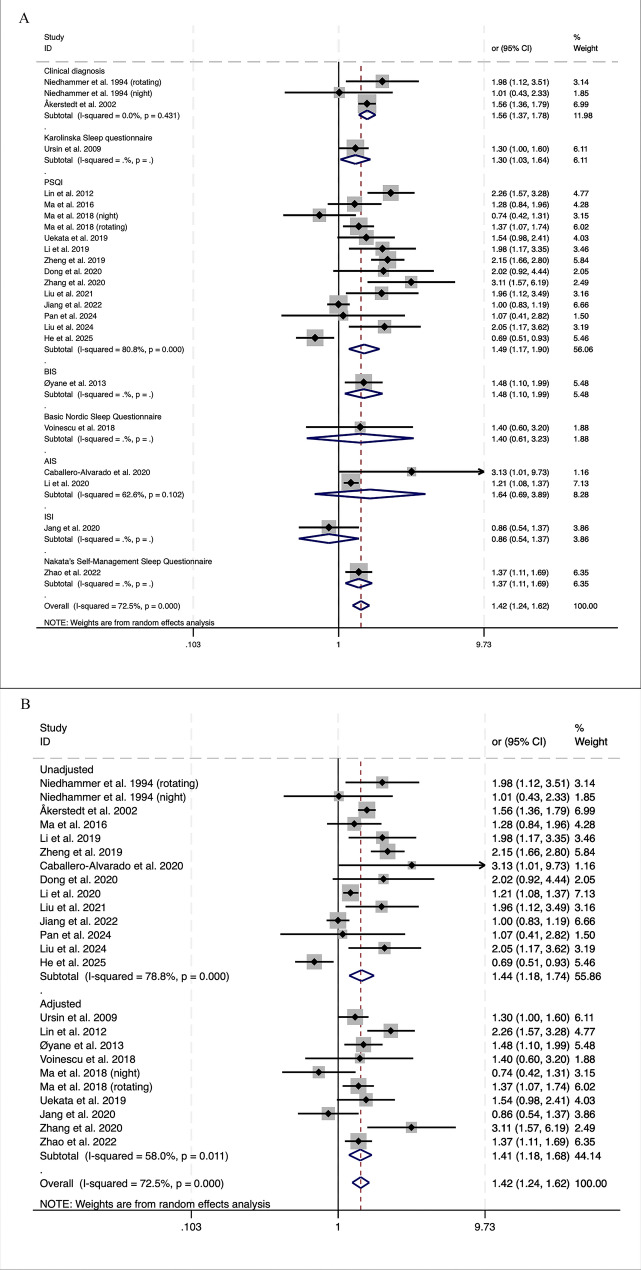
Subgroup analyses of the association between shift work and sleep disturbances by diagnostic method (**a**) and adjustment status (**b**). *Note.* OR = odds ratio; CI = confidence interval; PSQI = Pittsburgh Sleep Quality Index; BIS = Bergen Insomnia Scale; AIS = Athens Insomnia Scale; ISI = Insomnia Severity Index; Adjusted = ORs explicitly reported with covariate adjustment; Unadjusted = ORs not adjusted or reported as crude

Effect estimates varied according to the diagnostic method used to assess sleep disturbances (Fig. 6a). Studies based on clinical diagnosis reported a pooled OR of 1.56 (95% CI: 1.37–1.78) with no heterogeneity (I² = 0.0%). Studies using the PSQI reported an OR of 1.49 (95% CI: 1.17–1.90) with substantial heterogeneity (I² = 80.8%), whereas studies using the AIS showed an OR of 1.64 (95% CI: 0.69–3.89) with moderate heterogeneity (I² = 62.6%). Single-study subgroups, such as BIS, Basic Nordic Sleep Questionnaire (BNSQ), and Karolinska Sleep Questionnaire (KSQ), showed variable effect estimates.

#### Subgroup analyses of shift work and sleep disturbances by adjustment status

Associations between shift work and sleep disturbances were observed regardless of covariate adjustment status (Fig. 6b). The pooled OR was 1.44 (95% CI: 1.18–1.74) for studies reporting unadjusted estimates and 1.41 (95% CI: 1.18–1.68) for studies reporting adjusted estimates. Heterogeneity was substantial among unadjusted estimates (I² = 78.8%) and moderate among adjusted estimates (I² = 58.0%).

### Sensitivity analysis

The sensitivity analysis was conducted using a random-effects model with a leave-one-out method. For each study omitted, the ORs ranged from 1.38 to 1.47, with 95% CIs consistently overlapping the pooled estimate of 1.42 (95% CI: 1.24–1.62). Leave-one-out sensitivity analysis confirmed that no single study materially influenced the pooled estimate. Detailed effect estimates for each omitted study are provided in Supplementary Table S7 and Figure S3.

### Certainty of evidence

Low-certainty evidence indicates that night shift work is associated with sleep disturbances, as summarized in Supplementary Table S8. The evidence was not downgraded for risk of bias, inconsistency, or imprecision, and no publication bias was detected. However, because all studies were observational and no upgrading factors (e.g., dose-response) were present, the overall certainty of evidence remained low.

## Discussion

### Key findings and interpretation

This systematic review and meta-analysis of 22 studies provides quantitative evidence that shift work is associated with increased odds of sleep disturbances (OR = 1.42, 95% CI: 1.24–1.62). Our findings consolidate prior inconsistent evidence [14–16], indicating that shift work may be a risk factor for impaired sleep in occupational populations.

The substantial heterogeneity observed (I^2^ = 72.5%) underscores the importance of examining effect modifiers. Stratification by type of sleep disturbance indicated elevated risks across all categories, suggesting a broad association between shift work and sleep health. This consistency implies that the underlying harm may stem less from specific sleep pathologies and more from a fundamental disruption of circadian rhythms, which impairs sleep architecture broadly. Further analysis revealed specific shift work characteristics associated with heightened risk. Rotating shifts conferred greater detriment than permanent night shifts. Notably, night shifts ≤ 8 h were linked to a higher odd than longer night shifts. This counterintuitive finding may imply that shorter, more frequent night shifts preclude stable circadian adaptation, resulting in chronic misalignment and compounded sleep debt [53, 54]. In addition, behavioral factors may also play a role, as workers with shorter night shifts may allocate non-working time to family responsibilities, study, or other activities rather than sleep, potentially exacerbating sleep disturbances. Among the occupational subgroups analyzed, healthcare professionals exhibited the highest observed association. This may partly reflect the combination of circadian disruption, high-stress workloads, and unpredictable schedules that are common in healthcare roles. However, similar risk factors may also be present in other occupations, and the observed differences largely reflect the distribution of available data rather than a direct comparison between healthcare and non-healthcare groups. These patterns can be explained through intertwined physiological and psychosocial pathways. Disruption of circadian rhythms impairs melatonin secretion and core body temperature regulation, while work-related stress and social misalignment inherent to shift work may further degrade sleep quality through irregular sleep-wake patterns and reduced recovery opportunities [2, 55, 56].

Furthermore, broader sociocultural and methodological factors appear to influence the association. Our cross-national comparisons indicate that shift work is associated with elevated odds of sleep disturbances in both East Asia (OR = 1.40) and Europe (OR = 1.49). The lower heterogeneity among European studies may reflect more standardized labor regulations and working conditions. In contrast, the greater variability observed in East Asia likely arises from diverse occupational structures, varying implementation of shift systems, and differing cultural norms regarding nighttime work. These regional patterns suggest that the sleep health burden associated with shift work is universal but modulated by macro-level institutional and cultural environments.

Heterogeneity was also partly attributable to the measurement instruments used to assess sleep disturbances. Studies using clinical diagnosis (OR = 1.56) or PSQI (OR = 1.49) reported consistently higher pooled estimates, whereas those using tools such as AIS, ISI, or BNSQ showed lower or more variable estimates. This discrepancy likely stems from differences in the conceptual focus, sensitivity to specific sleep dimensions, cross-cultural validation of these instruments, and limited evidence regarding their use specifically in shift-working populations [57]. Regarding the PSQI specifically, the variability in cut-off scores across studies (e.g., > 5, ≥6, ≥ 7, ≥8) may introduce measurement bias or differential misclassification, potentially affecting the magnitude of observed associations.

### Strengths and limitations

This review strengthens public health evidence through methodologically rigorous procedures, including a comprehensive multi-database search, manual reference screening, duplicate data extraction, and independent quality assessment. Importantly, the analysis progresses beyond confirming an association to identifying actionable intervention points. Extensive subgroup analyses pinpointed high-risk scenarios–specifically rotating shifts, healthcare occupations, and shorter (≤ 8 h) night shifts–thereby offering prioritized targets for occupational health programs. The robustness of the primary finding is supported by consistently similar pooled estimates from both unadjusted and adjusted studies, suggesting that the association is not merely an artifact of commonly measured confounders and strengthening the inference of an independent effect of shift work on sleep health at the population level.

Several limitations in the extant literature must be acknowledged. All included studies were cross-sectional, precluding causal inference and raising concerns about reverse causation and healthy worker selection bias. In particular, if individuals with poor sleep health tend to avoid or leave shift work, the observed associations may underestimate the true impact of shift work on sleep. Evidence for certain subgroup comparisons was limited to single studies, constraining the precision and generalizability of those estimates. For public health translation, heterogeneity in definitions of shift work, night-shift duration, and rotation patterns across studies limits our ability to prescribe specific, optimized shift schedules. Key potential confounders, such as chronotype and precise light-exposure metrics, were inconsistently reported, potentially leading to residual confounding. While self-reported sleep measures are practical for large-scale studies, they may not capture all clinical nuances. Moreover, our inclusion criteria focused on nocturnal sleep disturbances, which may not capture daytime sleep problems such as excessive daytime sleepiness or napping difficulties that are common among shift workers. This narrow definition may limit the generalizability of our findings to all sleep issues experienced in shift-working populations. Additionally, the geographical concentration of studies in East Asia may limit the generalizability of risk estimates to regions with differing labor policies. Nevertheless, the consistency of the positive association across diverse analyses indicates that the core public health issue is widespread.

### Practical implications and recommendations

This meta-analysis identifies robust and stratified associations with clear implications for occupational health. The significant link between shift work and sleep disturbances underscores a substantial public health burden. However, translating these findings into effective practice requires balancing biological evidence with socioeconomic realities.

Our findings suggest that interventions should prioritize shift schedule architecture. The elevated odd associated with rotating shifts strongly supports the broader adoption of scheduling principles designed to stabilize circadian rhythms, such as limiting consecutive night shifts and ensuring adequate recovery intervals. However, the observation that shorter (≤ 8 h) night shifts are associated with greater odd complicates the issue. It implies that merely reducing shift duration is insufficient and may even be detrimental if it leads to more frequent or poorly spaced night work. Therefore, policy discussions and workplace negotiations must consider not only shift length but also the overall roster pattern and opportunities for recovery.

The particularly strong association observed among healthcare workers highlights a sector where systemic change is urgently needed yet fraught with practical challenges. Recommendations for this population must account for the intense operational pressures in healthcare settings. While evidence supports implementing Fatigue Risk Management Systems (FRMS) and protected rest facilities, their success depends on adequate staffing, institutional commitment, and a culture that prioritizes worker recovery as integral to patient safety rather than in conflict with it.

Although structural changes are crucial, individual-level interventions also play a key role in supporting circadian health. Encouraging strategically timed light exposure and the use of blue-light filtering eyewear may help support circadian alignment in shift workers [58]. For workers with significant circadian misalignment, the professionally guided, timed use of melatonin may be considered [59, 60]. Moreover, broader health behaviors such as maintaining regular physical activity and consistent mealtimes are foundational for supporting circadian health [61].

## Conclusion

This systematic review and meta-analysis provides comprehensive evidence that shift work, particularly rotating shifts or short night shifts (≤ 8 h), is associated with higher odds of sleep disturbances among occupational populations, with the strongest association observed among healthcare professionals. Given the global prevalence of nonstandard work schedules, these findings suggest that it may be warranted to consider evidence-based interventions. Future efforts could prioritize evaluating structured shift schedules that promote circadian adaptation, alongside targeted sleep health programs for high-risk occupations, with the aim of mitigating this public health concern.

## Supplementary information

Supplementary Material 1.

Supplementary Material 2.

Supplementary Material 3.

Supplementary Material 4.

## Data Availability

The datasets generated and/or analyzed during the current study are available from the corresponding author on reasonable request.

## Abbreviations

OR: Odds ratio
CI: Confidence interval
I²: I-squared
AHRQ: The Agency for Healthcare Research and Quality
PRISMA: Preferred Reporting Items for Systematic Reviews and Meta-Analyses
MeSH: Medical Subject Headings
VIP: VIP Database for Chinese Technical Periodicals
CNKI: China National Knowledge Infrastructure
Wanfang Data: Wanfang Data Knowledge Service Platform
CBM: Chinese Biomedical Literature Database
NOS: Newcastle-Ottawa Scale
PSQI: Pittsburgh Sleep Quality Index
BIS: Bergen Insomnia Scale
AIS: Athens Insomnia Scale
ISCO-08: International Standard Classification of Occupations
BNSQ: Basic Nordic Sleep Questionnaire
KSQ: Karolinska Sleep Questionnaire
ISI: Insomnia Severity Index
NA: Not available
FRMS: Fatigue Risk Management Systems
