# Supplementary material for: Shift work and risk of sleep disturbances in occupational populations: a systematic review and meta-analysis
Source: BMC Public Health. 2026 May 6;26:2254. doi: 10.1186/s12889-026-27636-2 (PMC13430882; doi:10.1186/s12889-026-27636-2)
Supplement: Supplementary file 2 — Supplementary Material 2. [file 12889_2026_27636_MOESM2_ESM.pdf]

NIHR | National Institute for Health and Care Research

PROSPERO
International prospective register of systematic reviews

Shift work and risk of sleep disturbances in occupational populations: a systematic review and meta-analysis

Ying Wang, Pei-Chao Wang, Xue-Ning Wang, Lin Yi, Dong-Liang Diao

Citation
Ying Wang, Pei-Chao Wang, Xue-Ning Wang, Lin Yi, Dong-Liang Diao. Shift work and risk of sleep disturbances in occupational populations: a systematic review and meta-analysis. PROSPERO 2026 CRD420261285200. Available from https://www.crd.york.ac.uk/PROSPERO/view/CRD420261285200.

REVIEW TITLE AND BASIC DETAILS

Review title
Shift work and risk of sleep disturbances in occupational populations: a systematic review and meta-analysis

Condition or domain being studied
Shift work; Sleep disturbances

Rationale for the review
Shift work is increasingly common worldwide and is known to disrupt circadian rhythms, potentially leading to sleep disturbances. Existing studies on this association are often limited to specific occupational groups or countries, and findings have been inconsistent, partly due to variations in study design, exposure definitions, and outcome measurements. A comprehensive synthesis across diverse working populations is lacking. This systematic review and meta-analysis aims to quantify the association between shift work and sleep disturbances, explore potential sources of heterogeneity, and provide evidence to inform occupational health strategies, shift scheduling practices, and future research.

Review objectives
To systematically review and meta-analyze observational studies examining the association between shift work and sleep disturbances in working populations, and to identify potential sources of heterogeneity by conducting subgroup analyses based on shift type, night-shift duration, occupation, country, region, and diagnostic method.

Keywords
Shift work; Sleep disturbances; Risk factors; occupational populations

Country
China

ELIGIBILITY CRITERIA

Population

Included
Occupational populations engaged in employment, including healthcare, industrial, transportation, service, and other occupational sectors, with data on shift work exposure and sleep disturbance outcomes.

Intervention(s) or exposure(s)

Included
Shift work; Rotating shift work

Exposure to shift work, including rotating shift works, permanent night shifts, or other nonstandard working schedules, in adult working populations. Studies must report sleep disturbance outcomes associated with shift work

Comparator(s) or control(s)
This review does not have any comparators

Study design
Only nonrandomized study types will be included.

Context
This review includes studies conducted in any occupational setting, including healthcare, industrial, transportation, service, and other work environments, where participants are engaged in shift work. Studies must report data on sleep disturbance outcomes. No restrictions are applied based on geographic region, workplace type, or cultural setting.

TIMELINE OF THE REVIEW

Date of first submission to PROSPERO
14 January 2026

Review timeline
Start date: 1 August 2025. End date: 31 March 2026.

Date of registration in PROSPERO
14 January 2026

AVAILABILITY OF FULL PROTOCOL

Availability of full protocol
A full protocol has been written but is not available because:

The full protocol has been completed but is not publicly available at this time because it has not yet been published or uploaded to a repository. It will be made available upon

publication of the review or as required by the journal.

SEARCHING AND SCREENING

Search for unpublished studies
Only published studies will be sought.

Main bibliographic databases that will be searched
The main databases to be searched are *CLIB – The Cochrane Library*, *Embase.com*, *PubMed*, *SCI – Science Citation Index* and *SSCI – Social Science Citation Index*.

Search language restrictions
There are no language restrictions.

Search date restrictions
Databases will be searched for articles published before 15 August 2025, there are no restrictions on search start date.

Other methods of identifying studies
Other studies will be identified by: *looking through all the articles that cite the papers included in the review ("snowballing" or forward citation searching)*.

Link to search strategy
A full search strategy is available in the full protocol as described in the *Availability of full protocol* section

Selection process
Studies will be screened independently by at least two people (or person/machine combination) with a process to resolve differences.

Other relevant information about searching and screening
None

DATA COLLECTION PROCESS

Data extraction from published articles and reports
Data will be extracted independently by at least two people (or person/machine combination) with a process to resolve differences.

Authors will be asked to provide any required data not available in published reports.

Study risk of bias or quality assessment
Risk of bias will be assessed using: *Newcastle–Ottawa*

Cohort and case–control studies were evaluated using the Newcastle–Ottawa Scale (NOS), while cross–sectional studies were assessed using the Agency for Healthcare Research and Quality (AHRQ) checklist.

Data will be assessed independently by at least two people (or person/machine combination) with a process to resolve differences.

Additional information will be sought from study investigators if required information is unclear or unavailable in the study publications/reports.

Reporting bias assessment
Risk of bias due to missing results will be assessed

Certainty assessment
The certainty (or confidence) of the evidence will be assessed using the GRADE approach. This method considers risk of bias, inconsistency, indirectness, imprecision, and publication bias across studies. Two reviewers will independently assess the certainty of findings, and any disagreements will be resolved by discussion or consultation with a third reviewer.

OUTCOMES TO BE ANALYSED

Main outcomes
The primary outcome is sleep disturbance among shift workers. Acceptable measurement instruments include validated questionnaires such as the Pittsburgh Sleep Quality Index (PSQI), Insomnia Severity Index (ISI), Karolinska Sleep Questionnaire (KSQ), and others. The effect measure to be used in synthesis is odds ratio (OR) with 95% confidence intervals.

Additional outcomes
There are no additional outcomes.

PLANNED DATA SYNTHESIS

Strategy for data synthesis
Formal synthesis is planned. Data will be pooled using either a fixed-effect or random-effects model depending on the level of heterogeneity, as assessed by the I^2 statistic. The primary effect measure will be odds ratios (ORs) with 95% confidence intervals for sleep disturbances associated with shift work. Substantial heterogeneity will be explored through subgroup analyses. Sensitivity analyses will be conducted using a leave-one-out approach. Publication bias will be assessed using funnel plots and Egger's test if ≥10 studies are included. When quantitative synthesis is not possible, findings will be summarized narratively.

CURRENT REVIEW STAGE

Stage of the review at this submission

Review stage	Started	Completed
Pilot work	✓	
Formal searching/study identification	✓	
Screening search results against inclusion criteria	✓	
Data extraction or receipt of IPD		

Review stage	Started	Completed
Risk of bias/quality assessment		
Data synthesis		

Review status
The review is currently planned or ongoing.

Publication of review results
Results of the review will be published.

REVIEW AFFILIATION, FUNDING AND PEER REVIEW

Review team members
Miss Ying Wang. ORCID: 0009-0001-6790-0236. Changchun University of Chinese Medicine. China.

No conflict of interest declared.

Mr Pei-Chao Wang. Affiliated Hospital of Shandong University of Traditional Chinese Medicine. China.

No conflict of interest declared.

Miss Xue-Ning Wang. Heilongjiang University of Chinese Medicine. China.

No conflict of interest declared.

Mrs Lin Yi (review guarantor and contact) Affiliated Hospital of Shandong University of Traditional Chinese Medicine. China.

No conflict of interest declared.

Mr Dong-Liang Diao. Affiliated Hospital of Shandong University of Traditional Chinese Medicine. China.

No conflict of interest declared.

Named contact
Mrs Lin Yi (kiwi100862025@163.com). Affiliated Hospital of Shandong University of Traditional Chinese Medicine. China.

Review affiliation
Affiliated Hospital of Shandong University of Traditional Chinese Medicine.

Funding source
Review has no funding and no agreed support from an academic institution and is done in authors' own time.

Peer review
There has been no peer review of this planned review.

ADDITIONAL INFORMATION

Review conflict of interest

Declared individual interests are recorded under team member details.. No additional interests are recorded for this review.

Medical Subject Headings

Shift Work Schedule; Sleep Initiation and Maintenance Disorders

SIMILAR REVIEWS

Check for similar records already in PROSPERO

PROSPERO identified a number of existing PROSPERO records that were similar to this one (last check made on 14 January 2026). These are shown below along with the reasons given by that the review team for the reviews being different and/or proceeding.

- Shift work and risk of sleep disturbances in occupational populations: A systematic review and meta-analysis [published 16 November 2025] [CRD420251231846]. **The authors did not check this review**
- Effects of Shift Work on Cognitive and Motor Performance in Nurses: A Systematic Review and Meta-Analysis [published 24 February 2025] [CRD420250653132]. The review was judged **not to be similar**
- prevalence of shift work sleep disorder and associated factor in Ethiopia: A systematic review and meta-analysis [published 20 October 2025] [CRD420251163146]. The review was judged **not to be similar**
- Impact of rotating and night shift work on nurses' mental health and intention to leave the profession: a systematic review and meta-analysis [published 16 December 2025] [CRD420251266247]. The review was judged **not to be similar**

PROSPERO version history

- Version 1.0, published 14 Jan 2026

Disclaimer

The content of this record displays the information provided by the review team. PROSPERO does not peer review registration records or endorse their content.

PROSPERO accepts and posts the information provided in good faith; responsibility for record content rests with the review team. The guarantor for this record has affirmed that the information provided is truthful and that they understand that deliberate provision of inaccurate information may be construed as scientific misconduct.

PROSPERO does not accept any liability for the content provided in this record or for its use. Readers use the information provided in this record at their own risk.

Any enquiries about the record should be referred to the named review contact
