# Supplementary material for: Shift work and risk of sleep disturbances in occupational populations: a systematic review and meta-analysis
Source: BMC Public Health. 2026 May 6;26:2254. doi: 10.1186/s12889-026-27636-2 (PMC13430882; doi:10.1186/s12889-026-27636-2)
Supplement: Supplementary file 3 — Supplementary Material 3. [file 12889_2026_27636_MOESM3_ESM.docx]

**Contents**

[Availability of code 2](#_Toc233353568)

[Figure S1. Funnel plot of publication bias for studies assessing the association between shift work and sleep disturbances. 3](#_Toc668277954)

[Figure S2. Egger’s regression test for publication bias. 3](#_Toc408099068)

[Figure S3. Sensitivity analysis of the relationship between shift work and sleep disturbances. 4](#_Toc252696954)

**Availability of code**

gen lnor=ln(or)

gen lnlci=ln(lci)

gen lnuci=ln(uci)

#Forest plot

metan lnor lnlci lnuci, label(namevar=study) random effect(or) eform

#Subgroup analyses

metan lnor lnlci lnuci, label(namevar=study) random effect(or) eform by()

#Funnel plot

meta funnel or lci uci, ci

#egger test

gen se = (lnuci - lnlci) / (2*1.96)

metabias lnor se, egger graph

#Sensitivity analysis

metaninf lnor lnlci lnuci, random label(namevar=study) eform

**
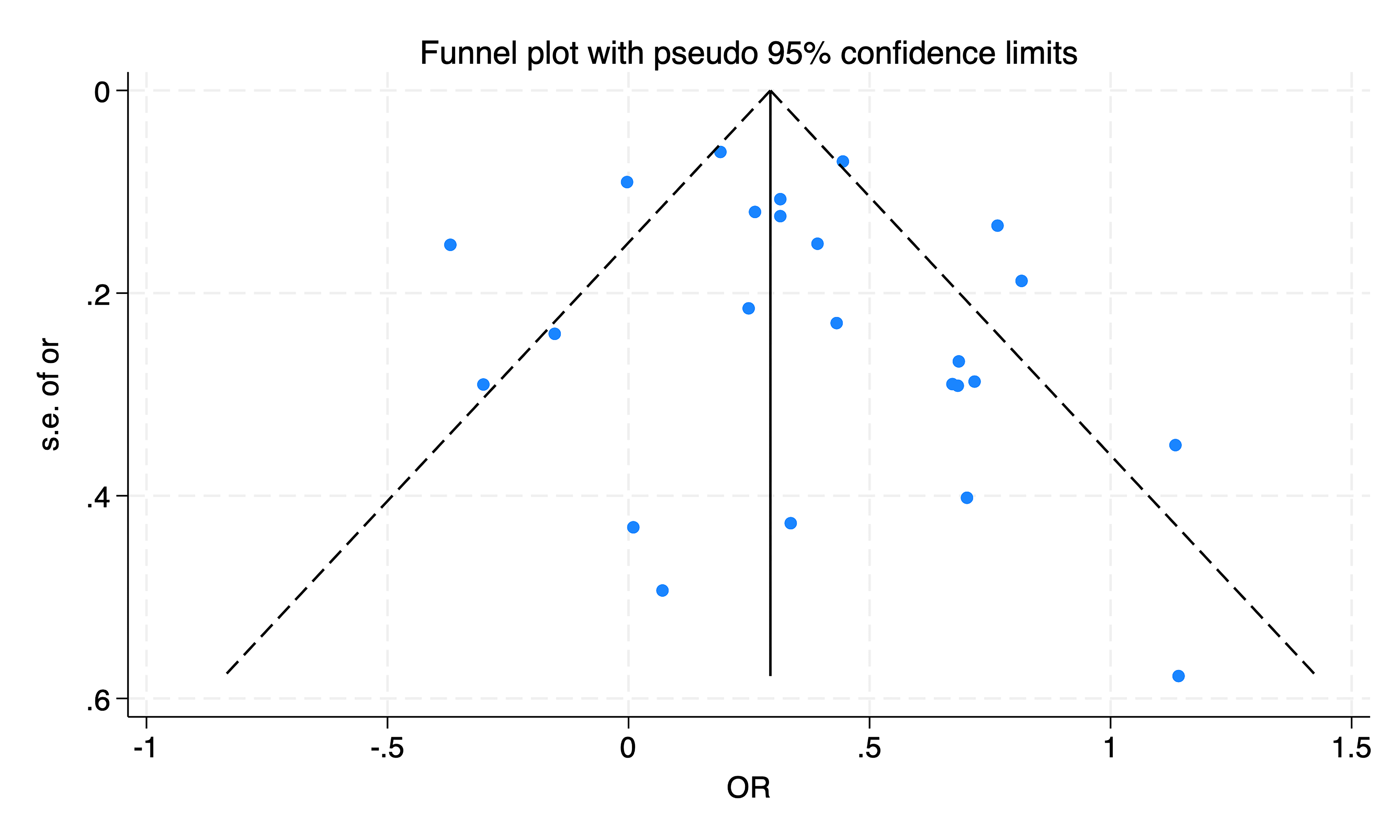
**

**Figure S1.** Funnel plot of publication bias for studies assessing the association between shift work and sleep disturbances.

**
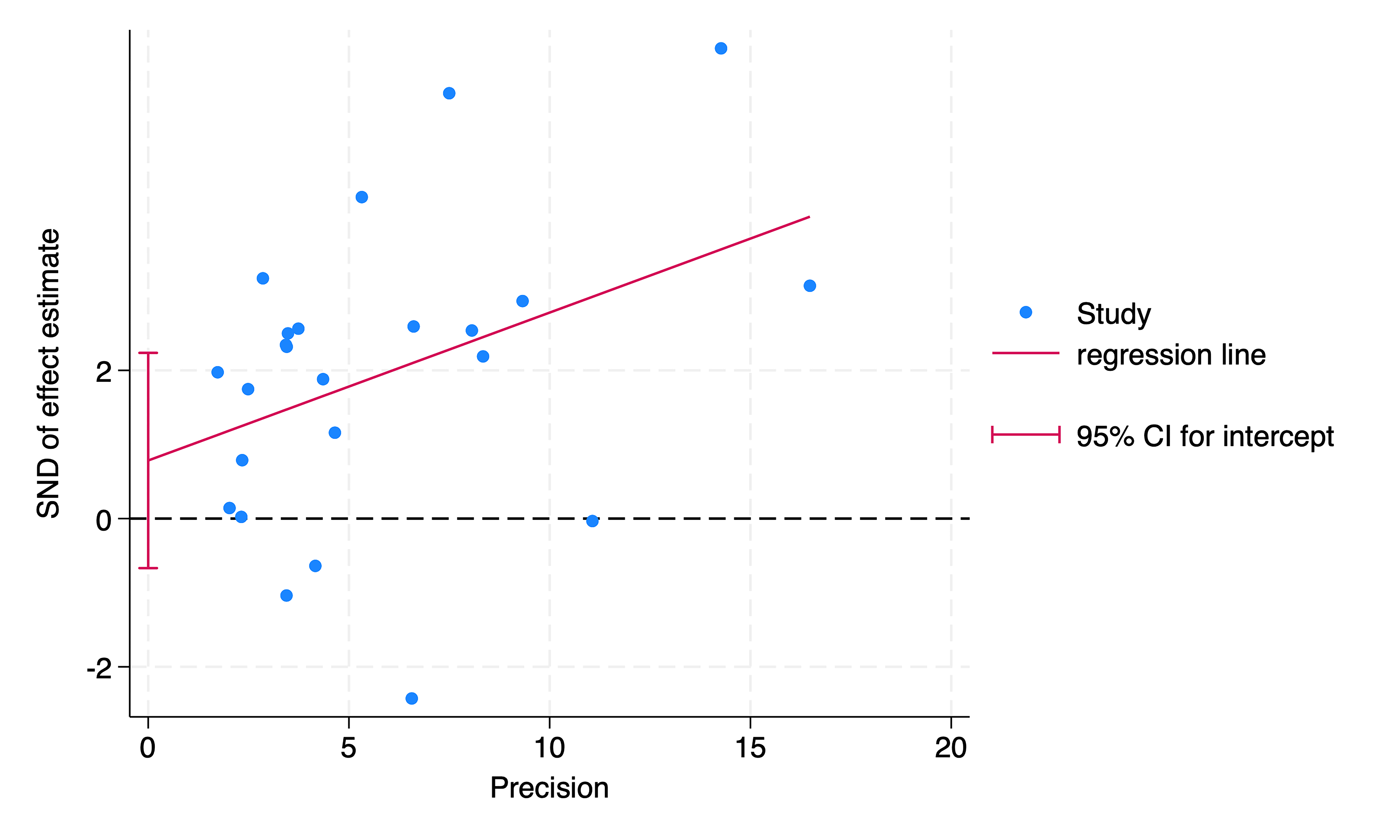
**

**Figure S2.** Egger’s regression test for publication bias.

**
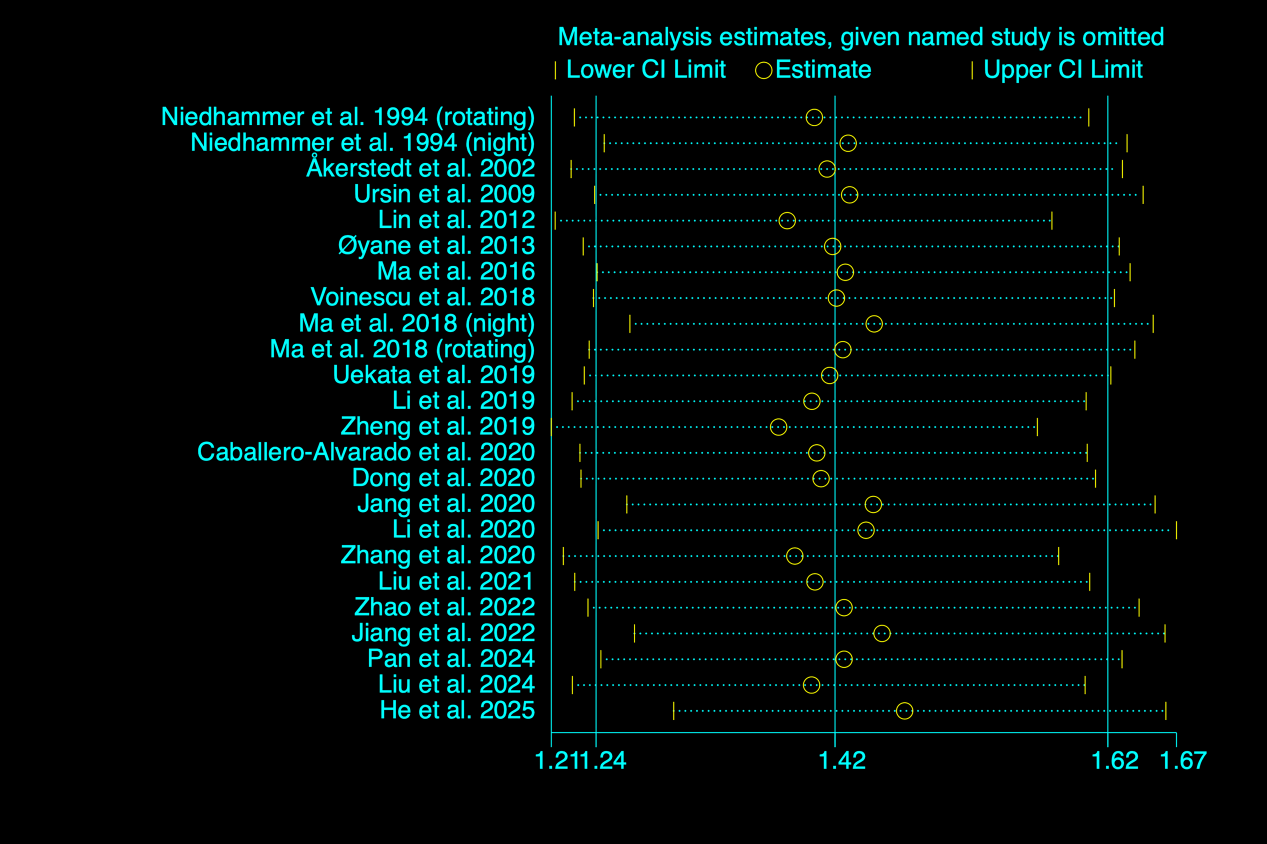
**

**Figure S3**. Sensitivity analysis of the relationship between shift work and sleep disturbances.

*Note.* CI = confidence interval.
