# Supplementary material for: Shift work and risk of sleep disturbances in occupational populations: a systematic review and meta-analysis
Source: BMC Public Health. 2026 May 6;26:2254. doi: 10.1186/s12889-026-27636-2 (PMC13430882; doi:10.1186/s12889-026-27636-2)
Supplement: Supplementary file 4 — Supplementary Material 4. [file 12889_2026_27636_MOESM4_ESM.docx]

**Contents**

[Table S1. Search strategy used for Pubmed database. 1](#_Toc1502535759)

[Table S2. Search strategy used for Web of Science database. 6](#_Toc858296440)

[Table S3. Search strategy used for Cochrane Libiary database. 7](#_Toc740610181)

[Table S4. Search strategy used for EMBASE database. 9](#_Toc620094055)

[Table S5. Search strategy used for CNKI, Wanfang Data, VIP and CBM database. 11](#_Toc182643494)

[Table S6. Risk of bias. 13](#_Toc935072095)

[Table S7. Sensitivity analysis. 14](#_Toc471371919)

[Table S8. Assessment of GRADE criteria (risk of bias, inconsistency, imprecision, and publication bias). 15](#_Toc280668850)

**Table S1**. Search strategy used for Pubmed database.

| Search number | Search Details | Results |
| --- | --- | --- |
| 7 | ("Sleep Initiation and Maintenance Disorders"[MeSH Terms] OR ("Disorders of Initiating and Maintaining Sleep"[Title/Abstract] OR "Sleep Initiation and Maintenance Disorders"[Title/Abstract] OR "DIMS"[Title/Abstract] OR "Sleeplessness"[Title/Abstract] OR "insomnia disorder"[Title/Abstract] OR "insomnia disorders"[Title/Abstract] OR "Insomnia"[Title/Abstract] OR "Insomnias"[Title/Abstract] OR "chronic insomnia"[Title/Abstract] OR "insomnia chronic"[Title/Abstract] OR "early awakening"[Title/Abstract] OR "awakening early"[Title/Abstract] OR "nonorganic insomnia"[Title/Abstract] OR (("insomnia s"[All Fields] OR "Sleep Initiation and Maintenance Disorders"[MeSH Terms] OR ("Sleep"[All Fields] AND "Initiation"[All Fields] AND "maintenance"[All Fields] AND "Disorders"[All Fields]) OR "Sleep Initiation and Maintenance Disorders"[All Fields] OR "Insomnia"[All Fields] OR "Insomnias"[All Fields]) AND "Nonorganic"[Title/Abstract]) OR "primary insomnia"[Title/Abstract] OR "insomnia primary"[Title/Abstract] OR "psychophysiological insomnia"[Title/Abstract] OR "insomnia psychophysiological"[Title/Abstract] OR "sleep initiation dysfunction"[Title/Abstract] OR (("dysfunctional"[All Fields] OR "dysfunctionals"[All Fields] OR "dysfunctioning"[All Fields] OR "Dysfunctions"[All Fields] OR "physiopathology"[MeSH Subheading] OR "physiopathology"[All Fields] OR "Dysfunction"[All Fields]) AND "sleep initiation"[Title/Abstract]) OR (("dysfunctional"[All Fields] OR "dysfunctionals"[All Fields] OR "dysfunctioning"[All Fields] OR "Dysfunctions"[All Fields] OR "physiopathology"[MeSH Subheading] OR "physiopathology"[All Fields] OR "Dysfunction"[All Fields]) AND "sleep initiation"[Title/Abstract]) OR ((("Sleep"[MeSH Terms] OR "Sleep"[All Fields] OR "sleeping"[All Fields] OR "sleeps"[All Fields] OR "sleep s"[All Fields]) AND ("initial"[All Fields] OR "initially"[All Fields] OR "initials"[All Fields] OR "initiate"[All Fields] OR "initiated"[All Fields] OR "initiates"[All Fields] OR "initiating"[All Fields] OR "Initiation"[All Fields] OR "initiations"[All Fields] OR "initiator"[All Fields] OR "initiators"[All Fields])) AND "Dysfunctions"[Title/Abstract]))) AND ("Shift Work Schedule"[MeSH Terms] OR ("Shift Work Schedule"[Title/Abstract] OR "schedule shift work"[Title/Abstract] OR "schedules shift work"[Title/Abstract] OR "work schedule shift"[Title/Abstract] OR "night shift work"[Title/Abstract] OR "shift work night"[Title/Abstract] OR "rotating shift work"[Title/Abstract] OR "shift work rotating"[Title/Abstract] OR "shift work"[Title/Abstract] OR "night shift"[Title/Abstract] OR "rotating shift"[Title/Abstract])) | 547 |
| 6 | "Shift Work Schedule"[MeSH Terms] OR "Shift Work Schedule"[Title/Abstract] OR "schedule shift work"[Title/Abstract] OR "schedules shift work"[Title/Abstract] OR "work schedule shift"[Title/Abstract] OR "night shift work"[Title/Abstract] OR "shift work night"[Title/Abstract] OR "rotating shift work"[Title/Abstract] OR "shift work rotating"[Title/Abstract] OR "shift work"[Title/Abstract] OR "night shift"[Title/Abstract] OR "rotating shift"[Title/Abstract] | 8,392 |
| 5 | "shift work schedule"[Title/Abstract] OR "schedule shift work"[Title/Abstract] OR "schedules shift work"[Title/Abstract] OR "work schedule shift"[Title/Abstract] OR "night shift work"[Title/Abstract] OR "shift work night"[Title/Abstract] OR "rotating shift work"[Title/Abstract] OR "shift work rotating"[Title/Abstract] OR "shift work"[Title/Abstract] OR "night shift"[Title/Abstract] OR "rotating shift"[Title/Abstract] | 8,103 |
| 4 | "Shift Work Schedule"[MeSH Terms] | 1,409 |
| 3 | "Sleep Initiation and Maintenance Disorders"[MeSH Terms] OR ("Disorders of Initiating and Maintaining Sleep"[Title/Abstract] OR "Sleep Initiation and Maintenance Disorders"[Title/Abstract] OR "DIMS"[Title/Abstract] OR "Sleeplessness"[Title/Abstract] OR "insomnia disorder"[Title/Abstract] OR "insomnia disorders"[Title/Abstract] OR "Insomnia"[Title/Abstract] OR "Insomnias"[Title/Abstract] OR "chronic insomnia"[Title/Abstract] OR "insomnia chronic"[Title/Abstract] OR "early awakening"[Title/Abstract] OR "awakening early"[Title/Abstract] OR "nonorganic insomnia"[Title/Abstract] OR (("insomnia s"[All Fields] OR "Sleep Initiation and Maintenance Disorders"[MeSH Terms] OR ("Sleep"[All Fields] AND "Initiation"[All Fields] AND "maintenance"[All Fields] AND "Disorders"[All Fields]) OR "Sleep Initiation and Maintenance Disorders"[All Fields] OR "Insomnia"[All Fields] OR "Insomnias"[All Fields]) AND "Nonorganic"[Title/Abstract]) OR "primary insomnia"[Title/Abstract] OR "insomnia primary"[Title/Abstract] OR "psychophysiological insomnia"[Title/Abstract] OR "insomnia psychophysiological"[Title/Abstract] OR "sleep initiation dysfunction"[Title/Abstract] OR (("dysfunctional"[All Fields] OR "dysfunctionals"[All Fields] OR "dysfunctioning"[All Fields] OR "Dysfunctions"[All Fields] OR "physiopathology"[MeSH Subheading] OR "physiopathology"[All Fields] OR "Dysfunction"[All Fields]) AND "sleep initiation"[Title/Abstract]) OR (("dysfunctional"[All Fields] OR "dysfunctionals"[All Fields] OR "dysfunctioning"[All Fields] OR "Dysfunctions"[All Fields] OR "physiopathology"[MeSH Subheading] OR "physiopathology"[All Fields] OR "Dysfunction"[All Fields]) AND "sleep initiation"[Title/Abstract]) OR ((("Sleep"[MeSH Terms] OR "Sleep"[All Fields] OR "sleeping"[All Fields] OR "sleeps"[All Fields] OR "sleep s"[All Fields]) AND ("initial"[All Fields] OR "initially"[All Fields] OR "initials"[All Fields] OR "initiate"[All Fields] OR "initiated"[All Fields] OR "initiates"[All Fields] OR "initiating"[All Fields] OR "Initiation"[All Fields] OR "initiations"[All Fields] OR "initiator"[All Fields] OR "initiators"[All Fields])) AND "Dysfunctions"[Title/Abstract])) | 42,839 |
| 2 | "Disorders of Initiating and Maintaining Sleep"[Title/Abstract] OR "Sleep Initiation and Maintenance Disorders"[Title/Abstract] OR "DIMS"[Title/Abstract] OR "Sleeplessness"[Title/Abstract] OR "insomnia disorder"[Title/Abstract] OR "insomnia disorders"[Title/Abstract] OR "Insomnia"[Title/Abstract] OR "Insomnias"[Title/Abstract] OR "chronic insomnia"[Title/Abstract] OR "insomnia chronic"[Title/Abstract] OR "early awakening"[Title/Abstract] OR "awakening early"[Title/Abstract] OR "nonorganic insomnia"[Title/Abstract] OR (("insomnia s"[All Fields] OR "Sleep Initiation and Maintenance Disorders"[MeSH Terms] OR ("Sleep"[All Fields] AND "Initiation"[All Fields] AND "maintenance"[All Fields] AND "Disorders"[All Fields]) OR "Sleep Initiation and Maintenance Disorders"[All Fields] OR "Insomnia"[All Fields] OR "Insomnias"[All Fields]) AND "Nonorganic"[Title/Abstract]) OR "primary insomnia"[Title/Abstract] OR "insomnia primary"[Title/Abstract] OR "psychophysiological insomnia"[Title/Abstract] OR "insomnia psychophysiological"[Title/Abstract] OR "sleep initiation dysfunction"[Title/Abstract] OR (("dysfunctional"[All Fields] OR "dysfunctionals"[All Fields] OR "dysfunctioning"[All Fields] OR "Dysfunctions"[All Fields] OR "physiopathology"[MeSH Subheading] OR "physiopathology"[All Fields] OR "Dysfunction"[All Fields]) AND "sleep initiation"[Title/Abstract]) OR (("dysfunctional"[All Fields] OR "dysfunctionals"[All Fields] OR "dysfunctioning"[All Fields] OR "Dysfunctions"[All Fields] OR "physiopathology"[MeSH Subheading] OR "physiopathology"[All Fields] OR "Dysfunction"[All Fields]) AND "sleep initiation"[Title/Abstract]) OR ((("Sleep"[MeSH Terms] OR "Sleep"[All Fields] OR "sleeping"[All Fields] OR "sleeps"[All Fields] OR "sleep s"[All Fields]) AND ("initial"[All Fields] OR "initially"[All Fields] OR "initials"[All Fields] OR "initiate"[All Fields] OR "initiated"[All Fields] OR "initiates"[All Fields] OR "initiating"[All Fields] OR "Initiation"[All Fields] OR "initiations"[All Fields] OR "initiator"[All Fields] OR "initiators"[All Fields])) AND "Dysfunctions"[Title/Abstract]) | 37,625 |
| 1 | "Sleep Initiation and Maintenance Disorders"[MeSH Terms] | 20,413 |

**Table S2**. Search strategy used for Web of Science database.

| # | Search Query | Results |
| --- | --- | --- |
| 1 | TS=(“Sleep Initiation and Maintenance Disorders” OR “Disorders of Initiating and Maintaining Sleep” OR DIMS OR Sleeplessness OR insomnia Disorder OR Insomnia Disorders OR Insomnia OR Insomnias OR Chronic Insomnia OR Insomnia, Chronic OR Early Awakening OR  Awakening, Early OR Nonorganic Insomnia OR Insomnia, Nonorganic OR Primary Insomnia OR Insomnia, Primary OR Psychophysiological Insomnia OR Insomnia, Psychophysiological OR Sleep Initiation Dysfunction OR Dysfunction, Sleep Initiation OR Dysfunctions, Sleep Initiation OR Sleep Initiation Dysfunctions) | 58310 |
| 2 | TS=(Shift Work Schedule OR Schedule, Shift Work OR Schedules, Shift Work OR Work Schedule, Shift OR Night Shift Work OR Shift Work, Night OR Rotating Shift Work OR Shift Work, Rotating OR Shift work OR night shift OR rotating shift) | 114726 |
| 3 | #1 AND #2 | 1253 |

**Table S3**. Search strategy used for Cochrane Libiary database.

| No. | Query | Results |
| --- | --- | --- |
| #38 | #24 AND #37 | 1182 |
| #37 | #25 OR #26 OR #27 OR #28 OR #29 OR #30 OR #31 OR #32 OR #33 OR #34 OR #35 OR #36 | 15541 |
| #36 | 'rotating shift':ab,ti | 826 |
| #35 | 'night shift':ab,ti | 5237 |
| #34 | 'shift work':ab,ti | 8057 |
| #33 | 'shift work, rotating':ab,ti | 7 |
| #32 | 'rotating shift work':ab,ti | 333 |
| #31 | 'shift work, night':ab,ti | 48 |
| #30 | 'night shift work':ab,ti | 1439 |
| #29 | 'work schedule, shift':ab,ti | 14 |
| #28 | 'schedules, shift work':ab,ti | 12 |
| #27 | 'schedule, shift work':ab,ti | 9 |
| #26 | 'shift work schedule':ab,ti | 164 |
| #25 | 'shift work'/exp | 8947 |
| #24 | #1 OR #2 OR #3 OR #4 OR #5 OR #6 OR #7 OR #8 OR #9 OR #10 OR #11 OR #12 OR #13 OR #14 OR #15 OR #16 OR #17 OR #18 OR #19 OR #20 OR #21 OR #22 OR #23 | 115001 |
| #23 | 'sleep initiation dysfunctions':ab,ti | 0 |
| #22 | 'dysfunctions, sleep initiation':ab,ti | 0 |
| #21 | 'dysfunction, sleep initiation':ab,ti | 0 |
| #20 | 'sleep initiation dysfunction':ab,ti | 0 |
| #19 | 'insomnia, psychophysiological':ab,ti | 7 |
| #18 | 'psychophysiological insomnia':ab,ti | 235 |
| #17 | 'insomnia, primary':ab,ti | 44 |
| #16 | 'primary insomnia':ab,ti | 1921 |
| #15 | 'insomnia, nonorganic':ab,ti | 1 |
| #14 | 'nonorganic insomnia':ab,ti | 39 |
| #13 | 'awakening, early':ab,ti | 35 |
| #12 | 'early awakening':ab,ti | 291 |
| #11 | 'insomnia, chronic':ab,ti | 97 |
| #10 | 'chronic insomnia':ab,ti | 3119 |
| #9 | 'insomnias':ab,ti | 270 |
| #8 | 'insomnia':ab,ti | 60463 |
| #7 | 'insomnia disorders':ab,ti | 157 |
| #6 | 'insomnia disorder':ab,ti | 2019 |
| #5 | 'sleeplessness':ab,ti | 1395 |
| #4 | 'dims':ab,ti | 677 |
| #3 | 'disorders of initiating and maintaining sleep':ab,ti | 146 |
| #2 | 'sleep initiation and maintenance disorders':ab,ti | 25 |
| #1 | 'insomnia'/exp | 104433 |

**Table S4**. Search strategy used for EMBASE database.

| ID | Search | Hits |
| --- | --- | --- |
| #1 | MeSH descriptor: [Sleep Initiation and Maintenance Disorders] explode all trees | 4025 |
| #2 | (“Sleep Initiation and Maintenance Disorders”):ti,ab,kw (Word variations have been searched) | 4037 |
| #3 | (“Disorders of Initiating and Maintaining Sleep”):ti,ab,kw (Word variations have been searched) | 56 |
| #4 | (DIMS):ti,ab,kw OR (Sleeplessness):ti,ab,kw OR (insomnia Disorder):ti,ab,kw OR (insomnia Disorders):ti,ab,kw OR (Insomnia):ti,ab,kw (Word variations have been searched) | 17409 |
| #5 | (Insomnias):ti,ab,kw OR (Chronic Insomnia):ti,ab,kw OR (Insomnia, Chronic):ti,ab,kw OR (Early Awakening):ti,ab,kw OR (Awakening, Early):ti,ab,kw (Word variations have been searched) | 16724 |
| #6 | (Nonorganic Insomnia):ti,ab,kw OR (Insomnia, Nonorganic):ti,ab,kw OR (Primary Insomnia):ti,ab,kw OR (Insomnia, Primary):ti,ab,kw OR (Psychophysiological Insomnia):ti,ab,kw (Word variations have been searched) | 7178 |
| #7 | (Insomnia, Psychophysiological):ti,ab,kw OR (Sleep Initiation Dysfunction):ti,ab,kw OR (Dysfunction, Sleep Initiation):ti,ab,kw OR (Dysfunctions, Sleep Initiation):ti,ab,kw OR (Sleep Initiation Dysfunctions):ti,ab,kw (Word variations have been searched) | 655 |
| #8 | #1 or #2 or #3 or #4 or #5 or #6 or #7 | 18773 |
| #9 | MeSH descriptor: [Shift Work Schedule] explode all trees | 70 |
| #10 | (Shift Work Schedule):ti,ab,kw OR (Schedule, Shift Work):ti,ab,kw OR (Schedules, Shift Work):ti,ab,kw OR (Work Schedule, Shift):ti,ab,kw OR (Night Shift Work):ti,ab,kw (Word variations have been searched) | 942 |
| #11 | (Shift Work, Night):ti,ab,kw OR (Rotating Shift Work):ti,ab,kw OR (Shift Work, Rotating):ti,ab,kw OR (Shift work):ti,ab,kw OR (night shift):ti,ab,kw (Word variations have been searched) | 3487 |
| #12 | (rotating shift):ti,ab,kw (Word variations have been searched) | 571 |
| #13 | #9 or #10 or #11 or #12 | 3867 |
| #14 | #8 and #13 | 330 |

**Table S5**. Search strategy used for CNKI, Wanfang Data, VIP and CBM database.

| Searching Strategies | Query | Results |
| --- | --- | --- |
| China National Knowledge Infrastructure (CNKI) | （主题：失眠 + 失眠症）OR（主题：慢性失眠 + 入睡和睡眠失调 + 入睡和睡眠障碍）OR（主题：不寐 + 睡眠起始功能障碍）OR（主题：早醒）OR（主题：原发性失眠）OR（主题：睡眠障碍）OR（主题：睡眠异常）OR（主题：睡眠不足）OR（主题：睡眠困难）OR（主题：入睡困难）AND（主题：轮班制）OR（主题：轮班）OR（主题：夜班）OR（主题：倒班）OR（主题：轮班工作） | 385 |
| Wanfang Data Knowledge Service Platform (Wanfang Data) | 主题:(失眠 OR 失眠症 OR 不寐 OR 早醒 OR 原发性失眠 OR 慢性失眠 OR 睡眠障碍 OR 睡眠异常 OR 睡眠不足 OR 睡眠困难 OR 入睡困难 OR 入睡和睡眠失调 OR 入睡和睡眠障碍 OR 睡眠起始功能障碍) AND 主题:(轮班制 OR 轮班 OR 夜班 OR 倒班 OR 轮班工作) | 852 |
| VIP Database for Chinese Technical Periodicals (VIP) | ((((((((((((((题名或关键词=失眠 OR 题名或关键词=失眠症) OR 题名或关键词=不寐) OR 题名或关键词=早醒) OR 题名或关键词=原发性失眠) OR 题名或关键词=慢性失眠) OR 题名或关键词=睡眠障碍) OR 题名或关键词=睡眠异常) OR 题名或关键词=睡眠不足) OR 题名或关键词=睡眠困难) OR 题名或关键词=入睡困难) OR 题名或关键词=入睡和睡眠失调) OR 题名或关键词=入睡和睡眠障碍) OR 题名或关键词=睡眠起始功能障碍) AND ((((题名或关键词=轮班制 OR 题名或关键词=轮班) OR 题名或关键词=夜班) OR 题名或关键词=倒班) OR 题名或关键词=轮班工作)) | 95 |
| Chinese Biomedical Literature Database (CBM) | (轮班制 OR 轮班 OR 夜班 OR 倒班 OR 轮班工作) AND (失眠 OR 失眠症 OR 不寐 OR 早醒 OR 原发性失眠 OR 慢性失眠 OR 睡眠障碍 OR 睡眠异常 OR 睡眠不足 OR 睡眠困难 OR 入睡困难 OR 入睡和睡眠失调 OR 入睡和睡眠障碍 OR 睡眠起始功能障碍) | 758 |

**Table S6**. Risk of bias.

| Std_Eff | Coefficient | Std. err. | t | P>t | [95% conf. interval] |
| --- | --- | --- | --- | --- | --- |
| slope | 0.1996374 | 0.1015022 | 1.97 | 0.062 | -0.0108653, 0.4101401 |
| bias | 0.7837546 | 0.7003097 | 1.12 | 0.275 | -0.6685989, 2.236108 |

*Note.* Number of studies = 24; Root MSE=1.895; Test of H0: no small-study effects, P = 0.275.

**Table S7**. Sensitivity analysis.

| Study omitted | Estimate | [95% Conf. Interval] |
| --- | --- | --- |
| Niedhammer et al. 1994 (rotating) | 1.4033295 | 1.226878, 1.6051584 |
| Niedhammer et al. 1994 (night) | 1.4281889 | 1.2488573, 1.6332721 |
| Åkerstedt et al. 2002 | 1.4127141 | 1.2244856, 1.6298771 |
| Ursin et al. 2009 | 1.4292876 | 1.2417779, 1.6451114 |
| Lin et al. 2012 | 1.3834006 | 1.2127733, 1.5780336 |
| Øyane et al. 2013 | 1.41682 | 1.2334032, 1.6275123 |
| Ma et al. 2016 | 1.4261539 | 1.2435985, 1.6355076 |
| Voinescu et al. 2018 | 1.4195769 | 1.2409098, 1.6239686 |
| Ma et al. 2018 (night) | 1.4473752 | 1.2677156, 1.652496 |
| Ma et al. 2018 (rotating) | 1.4243264 | 1.2377752, 1.6389937 |
| Uekata et al. 2019 | 1.4145895 | 1.2341841, 1.6213654 |
| Li et al. 2019 | 1.4015334 | 1.2252355, 1.6031985 |
| Zheng et al. 2019 | 1.3770568 | 1.209875, 1.56734 |
| Caballero-Alvarado et al. 2020 | 1.4051689 | 1.2309997, 1.6039807 |
| Dong et al. 2020 | 1.4082853 | 1.2317731, 1.6100913 |
| Jang et al. 2020 | 1.4467206 | 1.2654759, 1.6539236 |
| Li et al. 2020 | 1.4413542 | 1.2443138, 1.6695964 |
| Zhang et al. 2020 | 1.3889905 | 1.2187904, 1.5829585 |
| Liu et al. 2021 | 1.4037782 | 1.227169, 1.6058042 |
| Zhao et al. 2022 | 1.4251878 | 1.2369064, 1.6421293 |
| Jiang et al. 2022 | 1.4531376 | 1.2711165, 1.6612238 |
| Pan et al. 2024 | 1.425171 | 1.246387, 1.6296002 |
| Liu et al. 2024 | 1.401306 | 1.2254093, 1.6024512 |
| He et al. 2025 | 1.4697573 | 1.2999094, 1.6617976 |
| Combined | 1.4185737 | 1.2428337, 1.6191638 |

**Table S8**. Assessment of GRADE criteria (risk of bias, inconsistency, imprecision, and publication bias).

| Outcome | No. of participants (studies) | Risk of bias^1^ | Inconsistency^2^ | Imprecision^3^ | Publication bias^4^ | Other condersations^5^ | Odds Ratio  (95% CI) | Certainty of evidence |
| --- | --- | --- | --- | --- | --- | --- | --- | --- |
| Sleep disturbances among shift workers | 21677 (22) | Not serious | Not serious | Not serious | Not detected | none | 1.42 (1.24–1.62) | Low (⊕⊕◯◯) |

*Note.* CI = confidence interval.

^1^The evidence was downgraded by (−1) if the risk of bias was assessed as moderate according to the AHRQ tool. ^2^The evidence was downgraded by (−1) for inconsistency or heterogeneity, assessed based on differing effect estimates or I² statistics, if I² >75% and no plausible explanation was identified. ^3^The evidence was downgraded by (−1 or −2) for imprecision when the included studies had small sample sizes or wide confidence intervals. ^4^The evidence was downgraded by (−1) for publication bias, assessed by asymmetry of funnel plots or significant Egger’s test results. ^5^The certainty of evidence was upgraded by (+1) if a dose–response relationship was reported, and downgraded (−1) if the evidence was based on narrative synthesis or meta-analysis including only two studies.
